# Best match graphs and reconciliation of gene trees with species trees

**DOI:** 10.1007/s00285-020-01469-y

**Published:** 2020-01-30

**Authors:** Manuela Geiß, Marcos E. González Laffitte, Alitzel López Sánchez, Dulce I. Valdivia, Marc Hellmuth, Maribel Hernández Rosales, Peter F. Stadler

**Affiliations:** 1 Bioinformatics Group, Department of Computer Science, Interdisciplinary Center of Bioinformatics, University of Leipzig, Härtelstraße 16-18, 04107 Leipzig, Germany; 2 CONACYT-Instituto de Matemáticas, UNAM Juriquilla, Blvd. Juriquilla 3001, 76230 Juriquilla, Querétaro, QRO Mexico; 3 Centro de Ciencias Básicas, Universidad Autónoma de Aguascalientes, Av. Universidad 940, 20131 Aguascalientes, AGS México; 4 Instituto de Matemáticas, UNAM Juriquilla, Blvd. Juriquilla 3001, 76230 Juriquilla, Querétaro, QRO Mexico; 5 Institute of Mathematics and Computer Science, University of Greifswald, Walther-Rathenau-Straße 47, 17487 Greifswald, Germany; 6 Center for Bioinformatics, Saarland University, Building E 2.1, P.O. Box 151150, 66041 Saarbrücken, Germany; 7 German Centre for Integrative Biodiversity Research (iDiv) Halle-Jena-Leipzig, Leipzig, Germany; 8 Competence Center for Scalable Data Services and Solutions, Leipzig Research Center for Civilization Diseases, Leipzig University, Härtelstraße 16-18, 04107 Leipzig, Germany; 9 Max-Planck-Institute for Mathematics in the Sciences, Inselstraße 22, 04103 Leipzig, Germany; 10 Inst. f. Theoretical Chemistry, University of Vienna, Währingerstraße 17, 1090 Wien, Austria; 11 Facultad de Ciencias, Universidad National de Colombia, Bogotá, Colombia; 12 Santa Fe Institute, 1399 Hyde Park Rd., Santa Fe, NM 87501 USA

**Keywords:** Phylogenetic combinatorics, Colored digraph, Orthology, Horizontal gene transfer, 05C05, 05C62, 92B10

## Abstract

A wide variety of problems in computational biology, most notably the assessment of orthology, are solved with the help of reciprocal best matches. Using an evolutionary definition of best matches that captures the intuition behind the concept we clarify rigorously the relationships between reciprocal best matches, orthology, and evolutionary events under the assumption of duplication/loss scenarios. We show that the orthology graph is a subgraph of the reciprocal best match graph (RBMG). We furthermore give conditions under which an RBMG that is a cograph identifies the correct orthlogy relation. Using computer simulations we find that most false positive orthology assignments can be identified as so-called good quartets—and thus corrected—in the absence of horizontal transfer. Horizontal transfer, however, may introduce also false-negative orthology assignments.

**Electronic supplementary material:**

The online version of this article (10.1007/s00285-020-01469-y) contains supplementary material, which is available to authorized users.

## Introduction

The distinction between orthologous and paralogous genes has important consequences for gene annotation, comparative genomics, as well as molecular phylogenetics due to their close correlation with gene function (Koonin 2005). Orthologous genes, which derive from a speciation as their last common ancestor (Fitch 1970), usually have at least approximately equivalent functions (Gabaldón and Koonin 2013). Paralogs, in contrast, tend to have related, but clearly distinct functions (Studer and Robinson-Rechavi 2009; Innan and Kondrashov 2010; Altenhoff et al. 2012; Zallot et al. 2016). Phylogenetic studies strive to restrict their input data to one-to-one orthologs since these often evolve in an approximately clock-like fashion. In comparative genomics, orthologs serve as anchors for chromosome alignments and thus are an important basis for synteny-based methods (Sonnhammer et al. 2014).

Despite its practical importance, the mathematical interrelationships of empirical “pairwise best hits” on one hand, and reconciliations of gene and species trees on the other hand have remained largely unexplored. Practical workflows for orthology assignment directly use pairwise best hits as initial estimate of orthologous gene pairs. Many of the commonly used methods for orthology-identification, such as OrthoMCL (Li et al. 2003), ProteinOrtho (Lechner et al. 2014), OMA (Roth et al. 2008), or eggNOG (Jensen et al. 2008), belong to this class. Extensive benchmarking (Altenhoff et al. 2016; Nichio et al. 2017) has shown that these tools perform at least as well as methods such as Orthostrapper (Storm and Sonnhammer 2002), PHOG (Datta et al. 2009), EnsemblCompara (Vilella et al. 2009), or HOGENOM (Dufayard et al. 2005) that first independently reconstruct a gene tree *T* and a species tree *S* and then determine orthologous and paralogous genes.

**Fig. 1:**
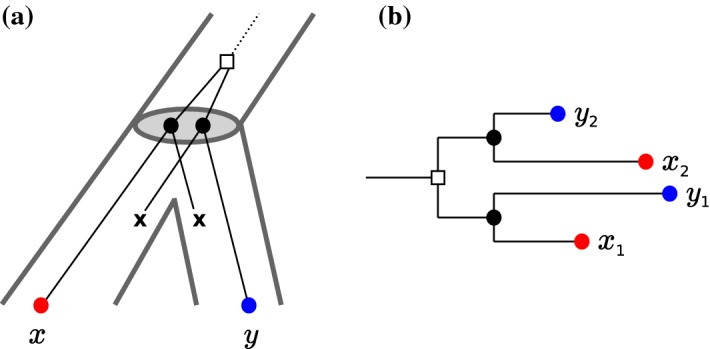
Pairwise best hits are not equivalent to orthology. **a** Complementary losses of ancient paralogs following a later speciation event leaves only a single member of the gene family in each species. Hence, *x* and *y* are reciprocal best matches but not orthologs since their last common ancestor by construction is a duplication event. **b** Lineage specific rate differences between paralogs cause discrepancies between best hits and best matches. Here, the branch length in the tree represents sequence dissimilarity. In this example, the species (indicated by the leaf color) retain copies of the two paralogs originating from a duplication event pre-dating the separation of red and blue. While the gene $x_2$ evolves faster in the red species, the situation is reversed for $y_2$ in the blue species. While $\{x_1,y_1\}$ and $\{x_2,y_2\}$ are orthologs and reciprocal best matches in the evolutionary sense, neither appears as a reciprocal best hit in terms of similarity (i.e., branch length). The only reciprocal best hit is $\{x_1,y_2\}$, which is neither a best match nor a pair of orthologs (color figure online)

The intuition behind the pairwise best hit approach is that a gene *y* in species *s* can only be an ortholog of a gene *x* in species *r* if *y* is the closest relative of *x* in *s* and *x* is at the same time the closest relative of *y* in *r*. Evolutionary relatedness is defined in terms of an – often unknown – phylogenetic tree *T*. The notion of a best match or closest relative thus is made precise by considering the last common ancestors in *T*: *y* is a best match for *x* if the least common ancestor ${{\,\mathrm{lca}\,}}_T(x,y)$ is not further away from *x* (and thus not closer to the root of the tree) than ${{\,\mathrm{lca}\,}}_T(x,y')$ for any other gene *y* in species *s*. This formally defines the *best match* relation studied in (Geiß et al. 2019a). The *reciprocal best match* relation identifies the pairs of genes that are mutually closest relatives between pairs of species, see (Geiß et al. 2019b).

Two approximations are introduced when pairwise best hit approaches are employed for orthology assessment. First, it is well known that two genes can be mutual closest relatives without being orthologs. The usual example is the complementary loss of ancestrally present paralogs following a gene duplication (Fig. 1a). Second, pairwise best hits as determined by sequence (dis)similiarity are not necessarily pairs of most closely related genes and *vice versa*, evolutionarily most closely related gene pairs do not necessarily appear as pairwise best hits (Fig. 1b).

We argue, therefore, that the relationship of pairwise best hits and orthology has to be understood in (at least) two conceptually and practically separate steps:

1. What is the relationship of pairwise best hits and reciprocal best matches?
2. What is the relation of reciprocal best matches and orthology?

In this contribution we focus on the second question, which is largely a mathematical problem. The main aim of the present contribution is to connect formal results on the structure of the orthology relation and the associated reconciliation maps and gene trees with recent results on the mathematical structure of (reciprocal) best match relations.

The first question, which is primarily a question of inference from data, is investigated in a companion paper (Stadler et al. 2020) that makes use of several of the mathematical results derived here. In a nutshell, the best hits inferred from estimates of genetic distances may differ from best matches whenever paralogs evolve with different rates in different species. In most situations this can be detected – and in most cases corrected – by considering quartets of genes $\{a,b_1,b_2,c\}$ from three different species, provided it is known that *c* is an outgroup to *a*, $b_1$, and $b_2$. Using the approximate additivity of empirical genetic distances, it can then be checked which one of the paralogs $b_1$ and $b_2$ is more closely related to *a*. The main practical difficulty is to ensure that *c* is correctly identified as outgroup.

Symbolic ultrametrics (Böcker and Dress 1998) and 2-structures (Ehrenfeucht and Rozenberg 1990a, b) provided a basis to show that orthology relations are essentially equivalent to cographs (Hellmuth et al. 2013, 2017; Hellmuth and Wieseke 2016). Moreover, in the absence of horizontal gene transfer (HGT), reconciliation maps for an event-labeled gene tree exist if and only if the species tree *S* displays all triples rooted in a speciation event that have leaves from three distinct species (Hernandez-Rosales et al. 2012; Hellmuth 2017). This shows that it is possible to infer species phylogenies from empirical estimates of orthology (Hellmuth et al. 2015; Lafond et al. 2016; Lafond and El-Mabrouk 2014; Dondi et al. 2017). Although it is possible to generalize many of the results, such as the characterization of reconciliation maps for event-labeled gene trees to scenarios with horizontal gene transfer (Nøjgaard et al. 2018; Hellmuth et al. 2019; Hellmuth 2017) this remains an active area of research.

Best matches as a mathematical structure have been studied only very recently. Geiß et al. (2019a) gave two alternative characterizations of best match digraphs and showed that they can be recognized in polynomial time. In particular, there is a unique least resolved tree for each best match digraph, which is displayed by the gene tree and can also be computed in polynomial time. Reciprocal best matches naturally appear as the symmetric part of these digraphs. Somewhat surprisingly, the undirected reciprocal best match graphs seem to have a much more difficult structure (Geiß et al. 2019b).

Although pairwise best hit methods do not attempt to explicitly construct the gene tree *T*, they still make the assumption that there is some underlying phylogeny for the provided homologous genes. The distinction of orthology and paralogy then amounts to assigning event labels (“speciation”, “duplication”, and possibly “HGT”) to the inner vertices of *T*. While it is true that any gene tree, and thus also any best match graph, can be reconciled with any species tree (Guigó et al. 1996; Page and Charleston 1997; Górecki and Tiuryn 2006), such a reconciliation may imply unrealistically many duplication and deletion events. In the extreme case, all inner vertices are duplication events before the first speciation. The root of the species tree then contains already a separate gene for each leaf of *T*. All the additional copies created by speciations therefore are eliminated again by subsequent loss events. More parsimonious reconciliations are thus usually modeled by minimizing the number duplication and loss events, reviewed e.g. by Doyon et al. (2011).

Moreover, the existence of reconciliation maps for *T* to some species tree cannot generally be ensured, if the event labels are given (Hernandez-Rosales et al. 2012; Hellmuth 2017). Hence, the best match relation (which constrains the gene tree (Geiß et al. 2019a)), the event labels, the existence of one or a particular reconcilation map, and the species tree depend on each other or at least do constrain each other. In this contribution we explore these dependencies in detail in the absence of horizontal gene transfer.

We show that, in this setting, the true orthology graph (TOG) is a subgraph of the reciprocal best match graph (RBMG). In other words, reciprocal best matches can only produce false positive orthology assignments as long as the evolution of a gene family proceeds via duplications, losses, and speciations. Computer simulations show that in broad parameter range the TOG and RBMG are very similar, proving an *a posteriori* justification for the use of reciprocal best matches in orthology estimation. In addition, we characterize a subset of the “false positive” edges in the RBMG that cannot be present in the TOG. Experimental results show that – using so-called good quartets – it is possible to remove nearly all false positive orthology assignments. Our aim here is to understand those sources of error and ambiguities in orthology detection that still persist even if reciprocal best matches are inferred with perfect accuracy. Therefore, all computer simulations reported here use perfect data as input. In a companion paper, we address the question how well reciprocal best matches can be inferred from (dis)similarity data, and what can be done to make this inital step more accurate. Finally, we discuss how these results can potentially be generalized to the case that the evolutionary scenarios contain HGT.

## Preliminaries

A *planted (phylogenetic) tree* is a rooted tree *T* with vertex set *V*(*T*) and edge set *E*(*T*) such that (i) the root $0_T$ has degree 1 and (ii) all inner vertices have degree $\deg _T(u)\ge 3$. We write *L*(*T*) for the leaves (not including $0_T$) and $V^0=V(T)\setminus (L(T)\cup \{0_T\})$ for the inner vertices (also not including $0_T$). To avoid trivial cases, we will always assume that $|L(T)|\ge 2$. The *conventional root*$\rho _T$ of *T* is the unique neighbor of $0_T$. The main reason for using planted phylogenetic trees instead of modeling phylogenetic trees simply as rooted trees, which is the much more common practice in the field, is that we will often need to refer to the time before the first branching event. Conceptually, it corresponds to explicitly representing an outgroup. For some vertex $v\in V(T)$, we denote by *T*(*v*) the subtree of *T* that is rooted in *v*. Its leaf set is *L*(*T*(*v*)).

On a rooted tree *T* we define the *ancestor order*: if *y* is a vertex of the unique path connecting *x* with the root $0_T$, we write $x\prec _T y$. As usual we write $x\preceq _T y$ if $x=y$ or $x\prec _T y$. In particular, the leaves are the minimal elements w.r.t. $\prec _T$, and we have $x\preceq 0_T$ for all $x\in V(T)$. This partial order is conveniently extended to the edge set by defining each edge to be located between its incident vertices, i.e., if $y\prec _T x$ and $e=xy$ is an edge, we set $y \prec _T e \prec _T x$. In this case, we write $e=xy$ to denote that *x* is closer to the root than *y*. If $e=xy\in E(T)$, we say that *y* is a *child* of *x*, in symbols $y\in \mathsf {child}(x)$, and *x* is the *parent* of *y* in *T*. We sometimes also write $y\succeq _T x$ instead of $x\preceq _T y$. Moreover, if $x\preceq _T y$ or $y\preceq _T x$ in *T*, then *x* and *y* are called *comparable*, otherwise the two vertices are *incomparable*.

For a non-empty subset of vertices $A\subseteq V$ of a rooted tree $T=(V,E)$, we define ${{\,\mathrm{lca}\,}}_T(A)$, the *last common ancestor of A*, to be the unique $\preceq _T$-minimal vertex of *T* that is an ancestor of every vertex in *A*. For simplicity we write ${{\,\mathrm{lca}\,}}_T(x_1,\dots ,x_k):={{\,\mathrm{lca}\,}}_T(\{x_1,\dots ,x_k\})$ for a set $A=\{x_1,\dots ,x_k\}$ of vertices. The definition of ${{\,\mathrm{lca}\,}}_T(A)$ is conveniently extended to edges by setting ${{\,\mathrm{lca}\,}}_T(x,e) :={{\,\mathrm{lca}\,}}_T(\{x\}\cup e)$ and ${{\,\mathrm{lca}\,}}_T(e,f) :={{\,\mathrm{lca}\,}}_T(e\cup f)$, where the edges $e,f\in E(T)$ are simply treated as sets of vertices. We note for later reference that ${{\,\mathrm{lca}\,}}(A\cup B)={{\,\mathrm{lca}\,}}({{\,\mathrm{lca}\,}}(A),{{\,\mathrm{lca}\,}}(B))$ holds for non-empty vertex sets *A*, *B* of a tree.

Binary trees on three leaves are called *triples*. We say that a triple *xy*|*z* is *displayed* in a rooted tree *T* if *x*, *y*,  and *z* are leaves in *T* and the path from *x* to *y* does not intersect the path from *z* to the root. The set of all triples that are displayed by the tree *T*, is denoted by *r*(*T*) and a triple set *R* is said to be *compatible* if there exists a tree *T* that displays *R*, i.e., $R\subseteq r(T)$.

Denote by *L*(*S*) a set of species and denote by $\sigma : L(T)\rightarrow L(S)$ the map that assigns to each gene $x\in L(T)$ a species $\sigma (x)\in L(S)$. A tree *T* together with such a map $\sigma$ is denoted by $(T,\sigma )$ and called *leaf-colored tree*.

### Definition 1

Let $(T,\sigma )$ be a leaf-colored tree. A leaf $y\in L(T)$ is a *best match* of the leaf $x\in L(T)$ if $\sigma (x)\ne \sigma (y)$ and ${{\,\mathrm{lca}\,}}(x,y)\preceq _T {{\,\mathrm{lca}\,}}(x,y')$ holds for all leaves $y'$ from species $\sigma (y')=\sigma (y)$. The leaves $x,y\in L(T)$ are *reciprocal best matches* if *y* is a best match for *x* and *x* is a best match for *y*.

The directed graph $\vec {G}(T,\sigma )$ with vertex set *L*(*T*), vertex-coloring $\sigma$, and edges defined by the best matches in $(T,\sigma )$ is known as *colored best match graph* (BMG) (Geiß et al. 2019a). The undirected graph $G(T,\sigma )$ with vertex set *L*(*T*), vertex-coloring $\sigma$, and edges defined by the reciprocal best matches in $(T,\sigma )$ is known as colored *reciprocal best match graph* (RBMG) (Geiß et al. 2019b). We sometimes write *n*-BMG, resp., *n*-RBMG to specify the number *n* of colors.

Throughout this contribution, $G=(V,E)$ and $\vec {G}=(V,\vec {E})$ denote simple undirected and simple directed graphs, respectively. We distinguish directed arcs (*x*, *y*) in a digraph $\vec {G}$ from edges *xy* in an undirected graph *G* or tree *T*. For an undirected graph *G* we denote by $N(x)=\{y \mid y \in V(G), xy\in E(G)\}$ the neighborhood of some vertex *x* in *G*. The *disjoint union* of two graphs $G=(V,E)$ and $H=(W,F)$ has vertex set  and edge set . Their *join* has again vertex set  and its edge set is given by . Thus the join of *G* and *H* is obtained by connecting every vertex of *G* to every vertex of *H*.

A class of undirected graphs that plays an important role in this contribution are *cographs*, which are recursively defined (Corneil et al. 1981):

### Definition 2

An undirected graph *G* is a cograph if one of the following conditions is satisfied:

1. $G=K_1$, the single-vertex graph,
2. $G= H {{\,\mathrm{\bowtie }\,}}H'$, where *H* and $H'$ are cographs,
, where *H* and $H'$ are cographs.

An undirected graph is a cograph if and only if it does not contain an induced $P_4$ (path on four vertices) (Corneil et al. 1981).

Every cograph *G* is associated with a set of phylogenetic trees ${\mathfrak {T}}_G$, usually referred to as the *cotrees* of *G*. Every cotree $T_G\in {\mathfrak {T}}_G$ corresponds to a possible recursive construction of *G*, where the cotree for the single-vertex graph $K_1$ is simply $K_1$. Since both the disjoint union and the join operation are associative, it is possible to join or unify two or more component cographs in a single construction step. The leaves of $T_G$ correspond to the vertices of *G*. Each interior vertex of $T_G$ corresponds to either a join or a disjoint union operation. Its child-subtrees, furthermore, are exactly the cotrees of the component cographs that are joined or disjointly unified, respectively. The event type associated with an inner vertex *u* will be denoted by $t_G(u)$. Each vertex *u* of $T_G$ can be associated with an induced subgraph $G[L(T_G(u))]$. A cotree $T_G$ is called *discriminating* if any two adjacent inner nodes represent different types of events. If $T_G\in {\mathfrak {T}}_G$ and $T_G'$ is obtained from $T_G$ by contracting a non-discriminating edge, i.e., an edge *uv* with $t_G(u)=t_G(v)$, then $T_G'\in {\mathfrak {T}}_G$. Every cograph has a unique discriminating cotree, which is obtained from any of its cotrees by contracting all non-discriminating edges (Corneil et al. 1981). We note, finally, that the discriminating cotree of *G* coincides with the modular decomposition tree of *G*.

## Reconciliation maps, event labelings, and orthology relations

A *gene* tree $T=(V,E)$ and a species tree $S=(W,F)$ are planted phylogenetics trees on a set of (extant) genes *L*(*T*) and species *L*(*S*), respectively. We assume that we know which gene comes from which species. Mathematically, this knowledge is represented by a map $\sigma :L(T)\rightarrow L(S)$ that assigns to each gene the species in whose genome it resides. Best match approaches start from a set of genes taken from a set of species. Hence, the “gene-species-association” is known. Moreover, species without sampled genes do not affect the best match graph and we can w.l.o.g. assume that $\sigma$ is a surjective map to avoid trivial cases. Note, however, that the definitions and results presented below naturally extend to general maps $\sigma$. We write $(T,\sigma )$ for a gene tree with given map $\sigma$.

An *evolutionary scenario* comprises a gene tree and a species tree together with a map $\mu$ from *T* to *S* that identifies the locations in the species tree *S* at which evolutionary events took place that are represented by the vertices of the gene tree *T*. The properties of the map $\mu$ of course depend on which types of evolutionary events are considered. In order to model evolutionary scenarios we assume that evolutionary events of different types do not occur concurrently. In particular, speciation and duplication are always strictly temporally ordered. Gene duplications therefore always occur along the edges of the species tree. Vertices on *T* that model speciation events, on the other hand, must be mapped to inner vertices of *S*.

From here on we will consider only Duplication/Loss secenarios, that is we explicitly exclude horizontal gene transfer (HGT). We will briefly discuss the effects of HGT in Sect. 8.

### Definition 3

*(Reconciliation Map)* Let $S=(W,F)$ and $T=(V,E)$ be two planted phylogenetic trees and let $\sigma :L(T)\rightarrow L(S)$ be a surjective map. A *reconciliation* from $(T,\sigma )$ to *S* is a map $\mu :V\rightarrow W\cup F$ satisfying

(R0) *Root Constraint.*$\mu (x) = 0_S$ if and only if $x = 0_T$.
(R1) *Leaf Constraint.* If $x\in L(T)$, then $\mu (x)=\sigma (x)$.
(R2) *Ancestor Preservation.*$x\prec _{T} y$ implies $\mu (x)\preceq _S \mu (y)$.
(R3) *Speciation Constraints.* Suppose $\mu (x)\in W^0$.
  (i) $\mu (x) = {{\,\mathrm{lca}\,}}_S(\mu (v'),\mu (v''))$ for at least two distinct children $v',v''$ of *x* in *T*.
  (ii) $\mu (v')$ and $\mu (v'')$ are incomparable in *S* for any two distinct children $v'$ and $v''$ of *x* in *T*.

Several alternative definitions of reconciliation maps for Duplication/Loss scenarios have been proposed in the literature, many of which have been shown to be equivalent. Nevertheless, we add yet another one because earlier variants do not clearly separate conditions pertaining to the structural congruence of gene tree and species tree (Axioms (R0), (R1), and (R2)) from conditions that (implicitly) distinguish event types, here (R3.i) and (R3.ii). This axiom system also generalizes easily to situations with horizontal transfer as we shall see in Sect. 8. We proceed by showing that it is equivalent to axioms that are commonly used in the literature, see e.g. Górecki and Tiuryn (2006), Vernot et al. (2008), Doyon et al. (2011), Rusin et al. (2014), Hellmuth (2017), Nøjgaard et al. (2018), and the references therein.

### Lemma 1

Let $\mu$ be a map from $(T=(V,E), \sigma )$ to $S=(W,F)$ that satisfies (R0) and (R1). Then, $\mu$ satisfies Axioms (R2) and (R3) if and only if $\mu$ satisfies

(R2’) *Ancestor Constraint.*
  Suppose $$x,y\in V$$ with $$x\prec _{T} y$$.
  (i) If $$\mu (x), \mu (y) \in F$$, then $$\mu (x)\preceq _S \mu (y)$$,
  (ii) otherwise, i.e., at least one of $\mu (x)$ and $\mu (y)$ is contained in *W*, $\mu (x)\prec _S\mu (y)$.
  (R3’) *Inner Vertex Constraint.*
    If $$\mu (x)\in W^0$$, then
    (i) $$\mu (x) = {{\,\mathrm{lca}\,}}_S(\sigma (L(T(x))))$$ and
    (ii) $\mu (v')$ and $\mu (v'')$ are incomparable in *S* for any two distinct children $v'$ and $v''$ of *x* in *T*.

### Proof

Assume first that (R2) and (R3) are satisfied for $\mu$.

Then property (R2’.i) is satisfied since it is the restriction of (R2) to $\mu (x),\mu (y)\in F$.

To see that (R2’.ii) holds, let $x\prec _T y$ and $\mu (x)\in W$ or $\mu (y)\in W$. Assume first that $\mu (y)\in W$. Property (R2) implies $\mu (x)\preceq _S \mu (y)$. Let *v* be the child of *y* that lies on the path from *y* to *x* in *T*, i.e., $x\preceq _T v \prec _T y$. Assume for contradiction that $\mu (x) = \mu (y)$. By Property (R2) we have $\mu (x) = \mu (v) = \mu (y)$. For every other child $v'$ of *y*, Property (R2) implies $\mu (v') \preceq _S \mu (y)=\mu (v)$. Thus, $\mu (v)$ and $\mu (v')$ are comparable; a contradiction to (R3.ii). Hence, $\mu (x) \prec _S \mu (y)$ and (R2’.ii) is satisfied. Now suppose $\mu (x)\in W$ and assume for contradiction that $\mu (x) = \mu (y)$. Thus $\mu (y)\in W$ and we can apply the same arguments as above to conclude that (R3.ii) is not satisfied. Hence, $\mu (x) \prec _S \mu (y)$ and (R2’.ii) is satisfied.

In order to show that (R3’) is satisfied, let $x\in V$ such that $\mu (x)\in W^0$. Properties (R3’.ii) and (R3.ii) are equivalent. It remains to show that (R3’.i) is satisfied. From (R2) we infer $\mu (y)\preceq _S\mu (x)$ for all $y\in \bigcup _{v\in \mathsf {child}(x)} L(T(v)) = L(T(x))$. Thus,

1 $$\begin{aligned} {{\,\mathrm{lca}\,}}_S(\sigma (L(T(x)))) \preceq \mu (x). \end{aligned}$$

Property (R3.i) implies that there are two distinct children $v',v''\in \mathsf {child}(x)$ with $\mu (x) = {{\,\mathrm{lca}\,}}_S(\mu (v'),\mu (v''))$. Again using (R3.ii), we know that the images $\mu (v')$ and $\mu (v'')$ are incomparable in *S*. The latter together with $\mu (y) \preceq _S \mu (v')$ for all $y\in L(T(v'))$ and $\mu (y') \preceq _S \mu (v'')$ for all $y'\in L(T(v''))$ implies

$$\begin{aligned} {{\,\mathrm{lca}\,}}_S(\mu (v'),\mu (v'')) = {{\,\mathrm{lca}\,}}_S(\sigma (L(T(v'))) \cup \sigma (L(T(v'')))) \preceq _S {{\,\mathrm{lca}\,}}_S(\sigma (L(T(x)))). \end{aligned}$$

In summary, ${{\,\mathrm{lca}\,}}_S(\sigma (L(T(x))))\preceq _S \mu (x) = {{\,\mathrm{lca}\,}}_S(\mu (v'),\mu (v'')) \preceq _S {{\,\mathrm{lca}\,}}_S(\sigma (L(T(x))))$ implies that $\mu (x) = {{\,\mathrm{lca}\,}}_S(\sigma (L(T(x))))$ and Property (R3’.i) is satisfied.

Therefore, (R2) and (R3) imply (R2’) and (R3’).

Conversely, assume now that (R2’) and (R3’) are satisfied for $\mu$. Clearly (R2’) implies (R2), and (R3’.ii) implies (R3.ii). It remains to show that (R3.i) is satisfied. Let $\mu (x)\in W^0$. By (R2’.ii) we have $\mu (x) \succ _S \mu (v_i)$ for all children $v_i\in \mathsf {child}(x) =\{v_1,\dots ,v_k\}$, $k\ge 2$. Therefore, $\mu (x) \succeq _S {{\,\mathrm{lca}\,}}_S(\mu (v_1), \dots , \mu (v_k))$. By (R3’.ii), the images $\mu (v_1), \dots , \mu (v_k)$ are pairwise incomparable in *S*. The latter and (R2’.i) imply ${{\,\mathrm{lca}\,}}_S(\mu (v_1), \dots , \mu (v_k)) = {{\,\mathrm{lca}\,}}_S(\bigcup _{i=1}^k\sigma (L(T(v_i)))) = {{\,\mathrm{lca}\,}}_S(\sigma (L(T(x)))) = \mu (x)$. It is easy to verify that ${{\,\mathrm{lca}\,}}_S(\mu (v_1), \dots , \mu (v_k)) = {{\,\mathrm{lca}\,}}_S(\mu (v'), \mu (v''))$ for at least two children $v',v''\in \mathsf {child}(x)$ is always satisfied. Hence, $\mu (x) = {{\,\mathrm{lca}\,}}_S(\mu (v'), \mu (v''))$ for some $v',v''\in \mathsf {child}(x)$ and thus, (R3.i) is satisfied.

Therefore, (R2’) and (R3’) imply (R2) and (R3). $\square$

A reconciliation map $\mu$ from $(T,\sigma )$ to a species tree *S* implicitly determines whether an inner node of *T* corresponds to a speciation or a duplication. Since we assume that distinct events are represented by distinct nodes of the gene tree, all duplication events are mapped to the edges of *S*. Vertices of *T* mapped to vertices of *S* thus represent speciations. We formalize this idea as follows:

### Definition 4

Given a reconciliation map $\mu$ from $(T,\sigma )$ to *S*, the *event labeling onT(determined by*$\mu$*)* is the map  given by:

The symbols $\circledcirc$ and $\odot$ identify the planted root $0_T$ and the leaves of *T*, respectively. Inner vertices are labeled $\square$ for duplication and  for speciation, respectively.

The event labeling $t_{\mu }$, by definition, is completely determined by a reconciliation map $\mu$. This raises two related questions: (1) which pattern of event labels can arise for reconciliation maps, and (2) what restriction does a given event labeling impose on the reconciliation map? To study these questions, we consider event-labeled trees (*T*, *t*) where the *event labeling* of *T* is a map  satisfying $t(0_T)=\circledcirc$, $t(x)=\odot$ for all $x\in L(T)$, and  for $x\in V^0(T)$. We interpret $\square$ as gene duplication event and  as speciation event.

A simple consequence of the Axioms (R0)-(R3) is the following result which is stated here for later reference. For the sake of completeness, we also provide a short proof.

### Lemma 2

Let $\mu$ be a reconciliation map from the leaf-colored tree $(T,\sigma )$ to $S=(W,F)$ and suppose that *x* is a vertex in *V*(*T*) with $\mu (x)\in W^0$. Then, $\sigma (L(T(v')))\cap \sigma (L(T(v''))) = \emptyset$ for any two distinct $v',v''\in \mathsf {child}(x)$.

### Proof

Assume for contradiction that there is a vertex $z\in \sigma (L(T(v')))\cap \sigma (L(T(v'')))$. By Condition (R2’), we have $\mu (x)\succ _S\mu (v')\succeq _S z$ and $\mu (x)\succ _S\mu (v'')\succeq _S z$. Thus, there is a path $P_1$ from $\mu (x)$ to *z* that contains $\mu (v')$ and a path $P_2$ from $\mu (x)$ to *z* that contains $\mu (v'')$. However, Condition (R3.ii) implies that $\mu (v')$ and $\mu (v'')$ are incomparable in *S*, that is, the subtree of *S* consisting of the two paths $P_1$ and $P_2$ must contain a cycle; a contradiction. $\square$

Lemma 2 has a simple interpretation: Since $\mu (x)\in W^0$, we have , i.e., *x* represents a speciation. The lemma thus states that any two subtrees of *T* rooted in distinct children of a speciation event are composed of genes from disjoint sets of species. It suggests the following

### Definition 5

An event labeling  is *well-formed* if  implies that $\sigma (L(T(v')))\cap \sigma (L(T(v''))) = \emptyset$ for any two distinct $v',v''\in \mathsf {child}(x)$.

Lemma 2 suggests to ask for a characterization of the event maps *t* for a given leaf-labeled tree $(T,\sigma )$ for which $(T,t,\sigma )$ admits a reconciliation map to some species tree. Definition 5 suggests to start by considering among the well-formed event labelings the one that designates every vertex of *T* that is not identified as a duplication because it violates Lemma 2.

### Definition 6

Let $(T,\sigma )$ be a leaf-labeled tree. The *extremal event labeling* of *T* is the map  defined for $u\in V(T)$ by

The extremal event labeling ${\hat{t}_T}$ is completely determined by $(T,\sigma )$. By construction, if $u\in V^0(T)$ is a duplication w.r.t. to the extremal event labeling ${\hat{t}_T}(u)=\square$, then $t(u)=\square$ for every well-formed event labeling *t* on $(T,\sigma )$.

It is a well-known result that it is always possible to reconcile a given pair of gene tree *T* and species tree *S*, see e.g. (Guigó et al. 1996; Page and Charleston 1997; Górecki and Tiuryn 2006). For convenience, we include a short direct proof of this fact.

### Lemma 3

For every tree $(T=(V,E),\sigma )$ there is a reconciliation map $\mu$ to any species tree *S* with leaf set $L(S)=\sigma (L(T))$.

### Proof

Let $S=(W,F)$ be an arbitrary species tree with leaf set *L*(*S*) and $e_0 = 0_S\rho _S$ be the unique root-edge of *S*. Set $\mu (0_T) = 0_S$ and $\mu (v) = \sigma (v)$ for all $v\in L(T)$. Thus, (R0) and (R1) are satisfied. Now, set $\mu (v) = e_0$ for all $v\in V^0 = V\setminus (L(T)\cup \{0_T\})$. Thus, $\mu (v)\notin W^0$ for all $v\in V^0$ and (R3) is trivially satisfied. Finally, for all $v, v'\in V^0$ and $y\in L(T)$ with $y\prec _T v\prec _T v '$ we have by construction of $\mu$ that $\mu (y)\prec _T \mu (v) = \mu (v')\prec _T\mu (0_T)$. Thus, (R2) is satisfied. $\square$

The reconciliation map $\mu$ constructed in the proof of Lemma 3 maps all inner vertices of the gene tree to the edge above the root of the species tree *S*, and hence $t_{\mu }(x)=\square$ for all inner vertices of *T*. The root of *S* already contains |*L*(*T*)| genes, one for each leaf of *T*. Every speciation event is therefore accompanied by complementary losses, and there are no further gene duplication events below the root.

The assignment of genes to species, i.e., a prescribed leaf coloring $\sigma$, however, implies further restrictions. In fact, it is not sufficient to require that the event labeling is well-formed. Instead, the simultaneous knowledge of $(T,t,\sigma )$ gives rise to stronger conditions on the species trees *S* with which $(T,t,\sigma )$ can be reconciled. Following (Hernandez-Rosales et al. 2012), we denote by ${\mathcal {S}}(T,t,\sigma )$ the set of triples $\sigma (a)\sigma (b)|\sigma (c)$ for which *ab*|*c* is a triple displayed by *T* such that (i) $\sigma (a)$, $\sigma (b)$, $\sigma (c)$ are pairwise distinct species and (ii) the root of the triple is a speciation event, i.e., . This set of triples characterizes the existence of a reconciliation map:

### Proposition 1

(Hernandez-Rosales et al. 2012; Hellmuth 2017) Given an leaf-labeled tree $(T,t,\sigma )$ with a well-formed event labeling *t* and a species tree *S* with $L(S)=\sigma (L(T))$, there is a reconciliation map $\mu :V(T)\rightarrow V(S)\cup E(S)$ such that the event labeling is consistent with Definition 4 if and only if *S* displays ${\mathcal {S}}(T,t,\sigma )$. In particular, $(T,t,\sigma )$ can be reconciled with a species tree if and only if ${\mathcal {S}}(T,t,\sigma )$ is a compatible set of triples.

**Fig. 2:**
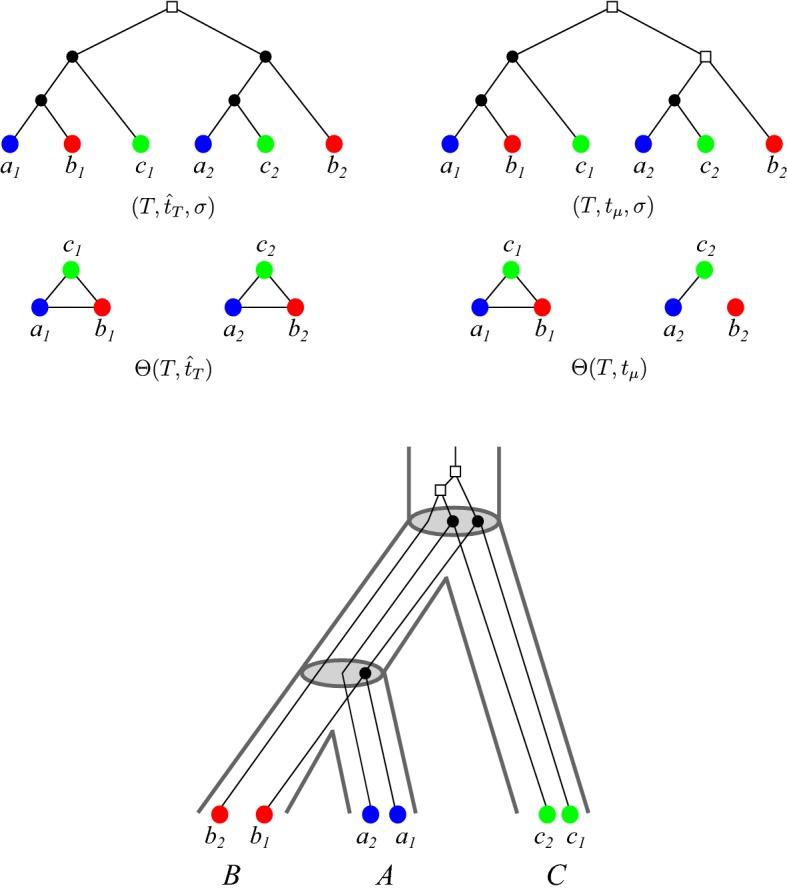
An example for $\varTheta (T,t_{\mu })\subset \varTheta (T,{\hat{t}_T})$. *Top Left:* A gene tree $(T,\sigma )$ with extremal event labeling ${\hat{t}_T}$, the corresponding orthology relation $\varTheta (T,{\hat{t}_T})$ and map $\sigma (a_i)=A$, $\sigma (b_i)=B$ and $\sigma (c_i)=C$, $i=1,2$. Here we obtain $AB|C, AC|B \in {\mathcal {S}}(T,{\hat{t}_T},\sigma )$ as conflicting species triples, making ${\mathcal {S}}(T,{\hat{t}_T},\sigma )$ incompatible. *Top Right and Bottom:* The same tree $(T,\sigma )$ with another event labeling $t_\mu$ defined by the reconciliation map $\mu$ from $(T,\sigma )$ to the (tube-like) species tree *S* as shown at the bottom. The map $\mu$ is given implicitly by drawing *T* into *S*. The corresponding orthology relation $\varTheta (T,t_\mu )$ is shown below $(T,t_{\mu }, \sigma )$. Clearly, since $\mu$ exists, ${\mathcal {S}}(T,t_{\mu },\sigma ) = \{AB|C\}$ is compatible (cf. Prop. 1) (color figure online)

An example for a $(T,t,\sigma )$ that does not admit a reconciliation map is given in Fig. 2 (top left). We note that the characterization in Proposition 1 can be evaluated in polynomial time (Hellmuth 2017).

The event labeling *t* on *T* defines the orthology relation:

### Definition 7

(Fitch 2000) Two distinct leaves $x,y\in L(T)$ are *orthologs (w.r.t.t)* if ; they are *paralogs* if $t({{\,\mathrm{lca}\,}}_T(x,y))=\square$.

**Fig. 3:**
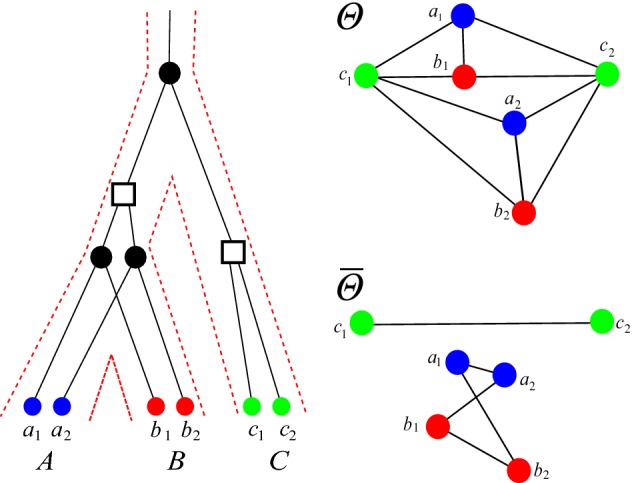
Orthology and paralogy relations are symmetric but not transitive. In this evolutionary scenario with two speciations () and two duplications ($\square$), the genes $a_1$ and $b_2$ are both orthologs of $c_1$ but not of each other. The leaves of the gene tree on the l.h.s. are colored corresponding to the three species *A*, *B*, and *C*. The orthology graph $\varTheta$ and its complement, the paralogy graph ${\overline{\varTheta }}$, are shown on the r.h.s (color figure online)

For completeness, we note that $t({{\,\mathrm{lca}\,}}_T(x,y))=\odot$ if and only $x=y$, and $0_T$ is never the ${{\,\mathrm{lca}\,}}$ of any of pair of leaves since the planted root $0_T$ has degree 1 by construction. We write $\varTheta (T,t)$ for the orthology relation obtained from (*T*, *t*), i.e., the set of all unordered pairs $\{x,y\}$ of orthologous genes in *L*(*T*). For convenience we will not distinguish between the irreflexive, symmetric binary relation $\varTheta (T,t)$ and the graph with vertex set *L*(*T*) and edge set $\varTheta (T,t)$. Naturally, we say that an arbitrary relation $\varTheta$ is an orthology relation if there is an event-labeled phylogenetic tree (*T*, *t*) such that $\varTheta =\varTheta (T,t)$. It is important to note that the orthology relation $\varTheta$ explicitly depends on the event labeling. Analogously, one can also define the *paralogy relation*${\overline{\varTheta }}$ by $t({{\,\mathrm{lca}\,}}_T(x,y))=\square$. Both orthology and paralogy are irreflexive and symmetric but not transitive, see Fig. 3. We note that orthology $\varTheta$ and paralogy ${\overline{\varTheta }}$ are complementary in the graph-theoretical sense, i.e., $\{x,y\}$ is contained in exactly one of $\varTheta$ or ${\overline{\varTheta }}$.

Based on the work of Böcker and Dress (1998) it has been shown by Hellmuth et al. (2013) that valid orthology relations are exactly cographs:

### Proposition 2

An irreflexive, symmetric relation $\varTheta$ on *L* is an orthology relation if and only if it is a cograph. In this case, every cotree *T* of $\varTheta$ with an event labeling *t* assigning  to join operations and $\square$ to disjoint union operations satisfies $\varTheta =\varTheta (T,t)$.

There is a unique discriminating cotree $(T_{\varTheta },t_{\varTheta })$ for an orthology relation $\varTheta$, which is obtained from every other (non-discriminating) cotree (*T*, *t*) for $\varTheta$ by contracting the inner edges *uv* of *T* if and only if $t(u)=t(v)$ (Böcker and Dress 1998; Hellmuth et al. 2013).

It is natural then to ask under which conditions a given orthology relation $\varTheta$ is consistent with a leaf-labeled tree $(T,\sigma )$ in the sense that there is a reconcilation map $\mu$ from $(T,\sigma )$ to some species tree such that $\varTheta =\varTheta (T,t_{\mu })$. We first consider the special case $T=T_{\varTheta }$. As shown by Hellmuth and Wieseke (2016), it is possible to obtain the set of informative triples ${\mathcal {S}}(T_{\varTheta },t_{\varTheta },\sigma )$ directly from $\varTheta$ using the following rule:

$\sigma (a)\sigma (b)|\sigma (c)\in {\mathcal {S}}(T,t,\sigma )$ if and only if $\sigma (a),\sigma (b)$, and $\sigma (c)$ are pairwise different species and either

- $$(a,c),(b,c)\in \varTheta $$ and $$(a,b)\not \in \varTheta $$*or*
- $(a,c),(b,c),(a,b)\in \varTheta$ and there is a vertex $d\ne a,b,c$ with $(c,d)\in \varTheta$ and $(a,d),(b,d)\notin \varTheta$.

### Theorem 1

Let $\varTheta$ be a cograph with vertex set *L* and associated cotree $(T_{\varTheta },t_{\varTheta })$ with leaf set *L* and let $\sigma$ be a leaf coloring. Then there exists a reconciliation map $\mu$ from $(T_{\varTheta },t_{\varTheta },\sigma )$ to some species tree *S* if and only if (i) ${\mathcal {S}}(T_{\varTheta },t_{\varTheta },\sigma )$ is compatible and (ii) the cograph $(\varTheta ,\sigma )$ is properly colored, i.e., for all $xy \in E(\varTheta )$ we have $\sigma (x)\ne \sigma (y)$.

### Proof

By Proposition 1, it is necessary and sufficient that (i) the set of informative triples is compatible and (ii) the event map $t_{\varTheta }$ is well-formed. Since $t_{\varTheta }$ is the event labeling of the co-tree, Condition (ii) amounts to requiring that the leaf set $L(T(v_i))$ have pairwise disjoint sets of colors $\sigma (L(T(v_i)))$ for all children $v_i\in \mathsf {child}(u)$ of every join node *u*. Since the join $\varTheta _i {{\,\mathrm{\bowtie }\,}}\varTheta _j$ of the two cographs associated with $T(v_i)$ and $T(v_j)$ introduces an edge *xy* for all $x\in L(T(v_i))$ and all $y\in L(T(v_j))$, the resulting graph can only be properly colored if $\sigma (L(T(v_i)))\cap \sigma (L(T(v_j)))=\emptyset$. On the other hand, every edge in $\varTheta$ is the result of a join operation, thus $(\varTheta ,\sigma )$ can only be well-colored if joins only appear between induced subgraphs with disjoint color sets. Thus $t_{\varTheta }$ is well-formed if and only if $\sigma$ is a proper vertex coloring for $\varTheta$. $\square$

Under the assumption that a reconciliation map $\mu$ exists for $(T,\sigma )$ to some species tree, the next results shows that the orthology relation $\varTheta (T,t_{\mu })$ is always a subgraph of the orthology relation $\varTheta (T,{\hat{t}_T})$ implied by $(T,\sigma )$ and its extremal labeling ${\hat{t}_T}$.

### Lemma 4

Let $(T,\sigma )$ be a leaf-labeled tree and $\mu$ a reconciliation map from $(T,\sigma )$ to some species tree *S*. Then $\varTheta (T,t_{\mu })\subseteq \varTheta (T,{\hat{t}_T})$.

### Proof

Let $u={{\,\mathrm{lca}\,}}_T(x,y)$ and suppose $xy\in \varTheta (T,t_{\mu })$. Then,  by definition of $\varTheta (T,t_{\mu })$, i.e., $\mu (u)\in V^0(S)$. Therefore, Lemma 2 implies $\sigma (L(T(v)))\cap \sigma (L(T(v')))=\emptyset$ for all $v,v'\in \mathsf {child}_T(u)$. Hence,  by definition of the extremal event labeling and thus $xy\in \varTheta (T,{\hat{t}_T})$. $\square$

The converse of Lemma 4 is generally not true, see Fig. 2 for an example. For later reference, we note the following result which is an immediate consequence of Lemma 4 due to the fact that orthology and paralogy relations are complementary.

### Corollary 1

Let $(T,\sigma )$ be a leaf-labeled tree and $\mu$ a reconciliation map from $(T,\sigma )$ to some species tree *S*. Then ${\overline{\varTheta }}(T,{\hat{t}_T})\subseteq {\overline{\varTheta }}(T,t_\mu )$.

Lemma 4, in particular, implies that none of the labelings $t_{\mu }$ (provided by any reconciliation map $\mu$) can yield more speciation events in *T*, than the extremal labeling ${\hat{t}_T}$. Moreover, it is easy to see that  always implies , while ${\hat{t}_T}(v) = \square$ implies $t_{\mu }(v) = \square$.

We briefly compare the formalism introduced here with the literature on maximum parsimony reconciliations. There, one considers reconciliation maps $\eta : V(T)\rightarrow V(S)$ that map duplication events in *T* also to vertices of *S*. The mapping $\eta$ is then interpreted in such a way that the duplication event *u* took place along an edge in *S* that is ancestral to $\eta (u)$. The map $\eta$ in this setting does not completely determine the event labeling. The least common ancestor map

2 $$\begin{aligned} {\hat{\eta }}(v):= {{\,\mathrm{lca}\,}}_{S}(\sigma (L(T(v))))\,. \end{aligned}$$

corresponds to one of the “most parsimonious reconciliations” (Górecki and Tiuryn 2006; Doyon et al. 2009) and can be obtained in polynomial time. A closely related reconciliation map can be defined in our setting. The *LCA-reconciliation map* introduced by Hellmuth (2017) satisfies the additional axiom

- (LCA) $\mu (u) = v{{\,\mathrm{lca}\,}}_S(\sigma (L(T(u))))\in E(S)$ for all $u\in V(T)$ with $t(u) = \square$, where *v* denotes the unique parent of ${{\,\mathrm{lca}\,}}_S(\sigma (L(T(u))))\in V(S)$ in *S*.

**Fig. 4:**
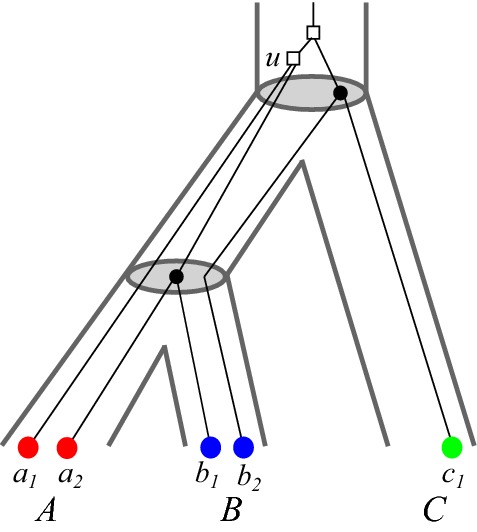
Reconciliation map $\mu$ from $(T,\sigma )$ to the (tube-like) species tree *S*. The map $\mu$ is given implicitly by drawing $(T,\sigma )$ into *S*. The map $\mu$ is not an LCA-reconciliation map since $\mu (u)$ does not map *u* to the edge $v{{\,\mathrm{lca}\,}}_S(A,B)\in E(S)$ where *v* denotes the unique parent of ${{\,\mathrm{lca}\,}}_S(A,B)$ in *S*. However, $t_{\mu }$ and the extremal map ${\hat{t}_T}$ coincide (color figure online)

The Axiom (LCA) is the analog of Eq. (2) for duplication vertices in *T*, which in our formalism are necessarily mapped to edges. For speciation events, the corresponding condition is expressed by (R3.i). Hellmuth (2017) showed that the existence of a reconciliation map from $(T,t,\sigma )$ implies also the existence of an LCA-reconciliation map. Figure 2 shows that an LCA-reconciliation map does not necessarily have ${\hat{t}_T}$ as its event labeling. Even if $t_{\mu }={\hat{t}_T}$, then $\mu$ is not necessarily an LCA-reconciliation map, see Fig. 4.

## Orthology and reciprocal best matches

In this section, we further clarify the relationship between the orthology relation and (reciprocal) best matches. As a main result, we find that the reciprocal best match graph contains any possible orthology relation.

### Lemma 5

If $(T,\sigma )$ with leaf set *L* explains the RBMG $(G,\sigma )$ and ${\hat{t}_T}$ is the extremal event labeling of $(T,\sigma )$, then $\varTheta (T,{\hat{t}_T})$ is a subgraph of the RBMG $G(T,\sigma )$.

### Proof

Consider a vertex $u\in V^0(T)$ with $\mathsf {child}(u) = \{u_1,\dots , u_k\}$. If ${\hat{t}_T}(u) = \square$, then none of the edges *xy* in *G* with $x\in L(T(u_i))$ and $y\in L(T(u_j))$, $1\le i<j\le k$ is contained in $\varTheta (T,{\hat{t}_T})$.

Now suppose . For $x\in L(T(u_i))$ and $y\in L(T(u_j))$ with $1\le i<j\le k$, we have $xy\in \varTheta (T,{\hat{t}_T})$ and, by construction of ${\hat{t}_T}$, $\sigma (x)\ne \sigma (y)$. In particular,  implies that all distinct children $u_i,u_j\in \mathsf {child}(u)$ satisfy $\sigma (L(T(u_i)))\cap \sigma (L(T(u_j))) =\emptyset$. Thus, ${{\,\mathrm{lca}\,}}_T(x,y) = u\preceq _T {{\,\mathrm{lca}\,}}_T(x',y)$ for all $x'\ne x$ with $\sigma (x') = \sigma (x)$ and ${{\,\mathrm{lca}\,}}_T(x,y) = u\preceq _T{{\,\mathrm{lca}\,}}_T(x,y')$ for all $y'\ne y$ with $\sigma (y') = \sigma (y)$, i.e., *x* and *y* are reciprocal best matches. Hence, $xy\in E(G)$ and thus $\varTheta (T,{\hat{t}_T})\subseteq G(T,\sigma )$. $\square$

Lemmas 4 and 5 immediately imply.

### Theorem 2

Let *T* and *S* be planted trees, $\sigma : L(T)\rightarrow L(S)$ a surjective map, and $\mu$ a reconciliation map from $(T,\sigma )$ to *S*. If $xy\in \varTheta (T,t_{\mu })$, then *x* and *y* are reciprocal best matches in $(T,\sigma )$.

### Observation 1

Reciprocal best matches therefore cannot produce false negative orthology assignments as long as the evolution of a gene family proceeds via duplications, losses, and speciations only.

The “false positive” edges in the RBMG compared to the orthology relation are the consequence of a particular class of duplication events:

### Theorem 3

Let $(T,t,\sigma )$ be a leaf- and event-labeled gene tree, $G(T,\sigma )$ and $\varTheta (T,t)$ its corresponding RBMG and orthology relation, respectively. Moreover, let $a,b\in L(T)$, $v:={{\,\mathrm{lca}\,}}_T(a,b)$, and $v_a,v_b\in \mathsf {child}_T(v)$ such that $a\preceq v_a\prec v$, $b\preceq v_b\prec v$. Then, $ab\in E(G(T,\sigma ))\setminus E(\varTheta (T,t))$ if and only if $t(v)=\square$ and $\sigma (a),\sigma (b)\in \sigma (L(T(v_a)))\mathbin {\triangle }\sigma (L(T(v_b)))$, where “$\mathbin {\triangle }$” denotes the usual symmetric set difference.

### Proof

Suppose first $ab\in E(G(T,\sigma ))\setminus E(\varTheta (T,t))$. By definition of $\varTheta (T,t)$, we immediately find $t(v)=\square$. Since $ab\in E(G(T,\sigma ))$, i.e., *a* and *b* are reciprocal best matches, it must hold $v\preceq _T{{\,\mathrm{lca}\,}}_T(a,b')$ for any $b'$ of color $\sigma (b)$. Hence, $\sigma (b)\notin \sigma (L(T(v_a)))$. Analogously, we conclude $\sigma (a)\notin \sigma (L(T(v_b)))$ and thus, $\sigma (a),\sigma (b)\in \sigma (L(T(v_a)))\mathbin {\triangle }\sigma (L(T(v_b)))$.

Conversely, assume $t(v)=\square$ and $\sigma (a),\sigma (b)\in \sigma (L(T(v_b)))\mathbin {\triangle }\sigma (L(T(v_a)))$. Since $t(v)=\square$, *a* and *b* cannot be orthologs, i.e., $ab\notin E(\varTheta (T,t))$. Moreover, $\sigma (a)\in \sigma (L(T(v_b)))\mathbin {\triangle }\sigma (L(T(v_a)))$ in particular implies $\sigma (a)\notin \sigma (L(T(v_b)))$ and therefore, $v\preceq _T {{\,\mathrm{lca}\,}}_T(a,b')$ for any $b'$ with $\sigma (b')=\sigma (b)$. Hence, *b* is a best match for *a* in species $\sigma (b)$. One similarly concludes that *a* is a best match for *b*. Hence, *a* and *b* are reciprocal best matches, which concludes the proof. $\square$

In practical application we usually do not know the event-labeled gene tree. It is possible, however, to compute the reciprocal best matches directly from sequence data. Therefore, it is of interest to investigate the relationship of reciprocal best match graphs and orthology relations.

### Definition 8

(Geiß et al. 2019b) A tree $(T,\sigma )$ is *least resolved* (w.r.t. the RBMG $G(T,\sigma )$ that it explains) if the contraction of any inner edge $e\in E(T)$ implies $G(T_e,\sigma )\ne G(T,\sigma )$.

Since $G(T,\sigma )$ is completely determined by $(T,\sigma )$ we can drop the reference to $G(T,\sigma )$ and often simply speak about a “least resolved tree”.

### Lemma 6

Let $(G,\sigma )$ be an RBMG that is explained by $(T,\sigma )$. If $(T,\sigma )$ is least resolved w.r.t. $(G,\sigma )$, then every inner edge $e=uv\in E(T)$ satisfies $\sigma (L(T(v)))\cap \sigma (L(T(u))\setminus L(T(v))) \ne \emptyset$.

### Proof

For contraposition, assume that there is an inner edge $e=uv\in E(T)$ with $\sigma (L(T(v)))\cap \sigma (L(T(u))\setminus L(T(v))) =\emptyset$. Hence, for all $x\in L(T(v))$ and $y\in L(T(u))\setminus L(T(v))$ we have ${{\,\mathrm{lca}\,}}_T(x,y) = u$ and $\sigma (x)=X\ne \sigma (y)=Y$. It is easy to see that all such *x* and *y* form a reciprocal best match and thus, $xy\in E(G)$. Clearly, *x* and *y* form also reciprocal best match in $(T_e,\sigma )$ and thus, each edge $xy\in E(G)$ with $x\in L(T(v))$ and $y\in L(T(u))\setminus L(T(v))$ is contained in $G(T_e,\sigma )$. Since we have not changed the relative ordering of the ${{\,\mathrm{lca}\,}}'s$ of the remaining vertices, all edges in *E*(*G*) are contained in $G(T_e,\sigma )$. $\square$

The converse of Lemma 6 is not necessarily true. As an example, consider an inner edge $e=uv\in E(T)$ with $\sigma (L(T(u))) = \sigma (L(T(v))) =\{c\}$. It is easy to see that *e* can be contracted.

Lemma 6 implies that if $(T,\sigma )$ is least resolved w.r.t. $G(T,\sigma )$ and $u\in V^0(T)$ such that *u* is incident to some other inner vertex $v\in \mathsf {child}(u)$, then there is a child $v'\ne v$ of *u* which satisfies $\sigma (L(T(v')))\cap \sigma (L(T(v)))\ne \emptyset$. By construction of ${\hat{t}_T}$ we have ${\hat{t}_T}(u) = \square$. The latter observation also implies the following:

### Corollary 2

Suppose that $(T,\sigma )$ is least resolved w.r.t. $G(T,\sigma )$ and let ${\hat{t}_T}$ be the extremal event labeling for $(T,\sigma )$. Then  if and only if all children of *u* are leaves that are from pairwise distinct species.

### Lemma 7

Let $(T,\sigma )$ be some least resolved tree (w.r.t. some RBMG) with extremal event map ${\hat{t}_T}$ and let *S*(*W*, *F*) be a species tree with $L(S)=\sigma (L(T))$. Then there is a reconciliation map $\mu : V(T)\rightarrow V(S)\cup E(S)$ such that $t_{\mu }={\hat{t}_T}$.

### Proof

By Cor. 2, every inner vertex *u* with  is only incident to leaves from pairwise distinct species. However, this implies that the set of informative species triples ${\mathcal {S}}(T,{\hat{t}_T},\sigma )$ is empty, and thus, compatible. Hence, Proposition 1 implies that there is a reconciliation map $\mu$ from $(T,{\hat{t}_T},\sigma )$ to any species tree *S*, defined by $\mu (0_T)=0_S$, $\mu (v) = 0_S\rho _S$ for every inner vertex $v\in V^0(T)$ that is incident to another inner vertex in *T*, and $\mu (v) = x ={{\,\mathrm{lca}\,}}_S(\sigma (L(T(v))))$ for any inner vertex *v* that is only incident to leaves that are from pairwise distinct species, and $\mu (v) = \sigma (v)$ for all leaves of *T*. By construction of $\mu$, we have ${\hat{t}_T}(u) = t_{\mu }(u)$ with $t_{\mu }(u)$ specified by Def. 4 for all $u\in V(T)$. $\square$

### Corollary 3

Let $(T,\sigma )$ be a least resolved tree explaining a co-RBMG $(G,\sigma )$. Then $(\varTheta (T,{\hat{t}_T}),\sigma )$ is a disjoint union of cliques.

### Proof

By Cor. 2 all children of a speciation node *u* w.r.t. ${\hat{t}_T}$ are leaves from pairwise distinct species. Thus the leaves *L*(*T*(*u*)) form a complete subgraph in $(\varTheta (T,{\hat{t}_T}),\sigma )$. On the other hand, no ancestor of *u* is a speciation, i.e., there is no edge *ab* with $a\in L(T(u))$ and $b\notin L(T(u))$. Thus $(\varTheta (T,{\hat{t}_T}),\sigma )$ is a disjoint union of the cliques formed by the *L*(*T*(*u*)) with  possibly together with isolated vertices that are not children of any speciation node in $(T,{\hat{t}_T})$. $\square$

**Fig. 5:**
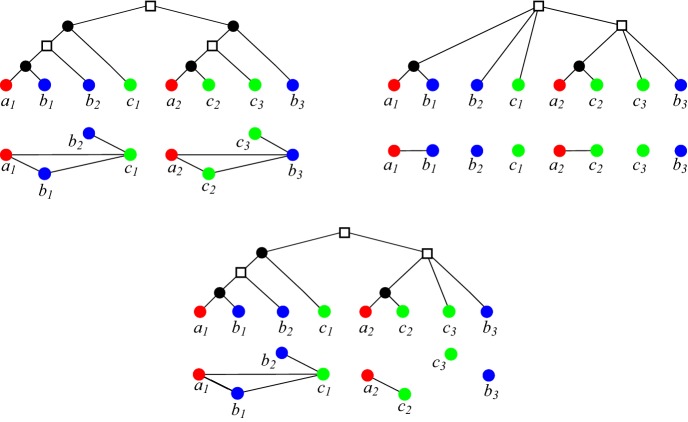
*Top Left:* A (discriminating) *hc*-cotree $(T^G_hc ,t_{hc },\sigma )$. Its corresponding *hc*-cograph $(G,\sigma ) = (\varTheta (T^G_hc ,t_{hc }),\sigma )$ is drawn below $(T^G_hc ,t_{hc },\sigma )$. In fact, Prop. 3 implies that $(G,\sigma )$ is an RBMG. *Top Right:* A tree $(T^*,{\hat{t}_T},\sigma )$ that is least resolved w.r.t. the RBMG $(G,\sigma )$ together with extremal labeling ${\hat{t}_T}$ and the resulting orthology relation $\varTheta (T^*,{\hat{t}_T})$, where $(T^*,{\hat{t}_T})$ is not discriminating. *Below:* A tree $(T,{\hat{t}_T},\sigma )$ together with extremal labeling ${\hat{t}_T}$ that explains the RBMG $(G,\sigma )$ but is not least resolved w.r.t. $(G,\sigma )$. The resulting orthology relation $\varTheta (T,{\hat{t}_T})$ is drawn below $(T,{\hat{t}_T},\sigma )$ (color figure online)

Suppose that we know the orthology relation $\varTheta (T,{\hat{t}_T})$ that is obtained from a least resolved tree $(T,\sigma )$ that explains the RBMG $(G,\sigma )$. Lemma 7 implies that there is always a reconciliation map $\mu$ from $(T,\sigma )$ to any species tree *S* with $L(S)=\sigma (L(T))$ such that ${\hat{t}_T}$ is determined by $\mu$ as in Def. 4. Now we can apply Theorem 2 to conclude that all orthologous pairs in $\varTheta (T,{\hat{t}_T})$ are reciprocal best matches. In other words, all complete subgraphs of $\varTheta (T,{\hat{t}_T})$ are also induced subgraphs of the underlying RBMG $(G,\sigma )$. Hence, $\varTheta (T,{\hat{t}_T})$ is obtained from $(G,\sigma )$ by removing edges such that the resulting graph is the disjoint union of cliques, see the top-right tree in Fig. 5 for an example. However, Fig. 5 also shows that many edges have to be removed to obtain $\varTheta (T,{\hat{t}_T})$.

This observation establishes the precise relationship of orthology detection and clustering, since (graph) clustering can be interpreted as the graph editing problem for disjoint unions of complete graphs (Böcker et al. 2011). In many orthology prediction tools, such as e.g. OMA (Roth et al. 2008), orthologs are summarized as *clusters of orthologous groups (COGs)* (Tatusov et al. 1997) that are obtained from reciprocal best matches.

The results above show that the RBMGs contain the orthology relation. Equivalently, RBMGs imply constraints on the event labeling. We also observe that the RBMGs cannot provide conclusive evidence regarding edges that *must* correspond to orthologous pairs. In the following sections we consider the constraints implied by the detailed structure of RBMGs or BMGs in more detail.

## Classification of RBMGs

The structure of RBMGs has been studied in extensive detail by Geiß et al. (2019b). Although we do not have an algorithmically useful complete characterization of RBMGs, there are partial results that can be used to identify different subclasses of RBMGs based on the structure of the connected components of the 3-colored subgraphs (Geiß et al. 2019b, Thm. 7). Let ${\mathscr {C}}(G,\sigma )$ be the set of the connected components of the induced subgraphs on three colors of an RBMG $(G,\sigma )$. Then every $(C,\sigma )\in {\mathscr {C}}(G,\sigma )$ is precisely of one of the three types (Geiß et al. 2019b, Thm. 5):

- **Type (A)**$(C,\sigma )$ contains a $K_3$ on three colors but no induced $P_4$.
- **Type (B)**$(C,\sigma )$ contains an induced $P_4$ on three colors whose endpoints have the same color, but no induced cycle $C_n$ on $n\ge 5$ vertices.
- **Type (C)**$(C,\sigma )$ contains an induced cycle $C_6$, called *hexagon*, such that any three consecutive vertices have pairwise distinct colors.

The graphs for which all $(C,\sigma )\in {\mathscr {C}}(G,\sigma )$ are of Type (A) are exactly the RBMGs that are cographs, or co-RBMGs for short (Geiß et al. 2019b, Thm. 8 and Remark 2). Intuitively, these have a close connection to orthology graphs because orthology graphs are cographs.

Connected components of Type (B) and Type (C), on the other hand, contain induced $P_4\hbox {s}$ and thus are neither cographs nor connected components of cographs. Obs. 1 implies that RBMGs that contain connected components of Type (B) and Type (C) introduce false positive edges into estimates of the orthology relation. In Sect. 6 below we will address the question to what extent and how such false-positives edges can be identified. We distinguish here co-RBMGs, *(B)-RBMGs*, and *(C)-RBMGs* depending on whether ${\mathscr {C}}(G,\sigma )$ contains only Type (A) components, at least one Type (B) but not Type (C) component, or at least one Type (C) component.

Co-RBMGs have a convenient structure that can be readily understood in terms of *hierarchically colored cographs* (*hc*-cographs) introduced by Geiß *et al.* (2019b, Sect. 7).

### Definition 9

An undirected colored graph $(G,\sigma )$ is a *hierarchically colored cograph (*hc*-cograph)* if

1. $(G,\sigma )=(K_1,\sigma )$, i.e., a colored vertex, or
2. $$(G,\sigma )= (H_1,\sigma _{H_1}) {{\,\mathrm{\bowtie }\,}}(H_2,\sigma _{H_2})$$ and $$\sigma (V(H_1))\cap \sigma (V(H_2))=\emptyset $$, or
and $$\sigma (V(H_1))\cap \sigma (V(H_2)) \in \{\sigma (V(H_1)),\sigma (V(H_2))\}$$,

where both $(H_1,\sigma _{H_1})$ and $(H_2,\sigma _{H_2})$ are *hc*-cographs and $\sigma (x)=\sigma _{H_i}(x)$ for any $x\in V(H_i)$ for $i\in \{1,2\}$.

Not all properly colored cographs are *hc*-cographs, see e.g. Geiß et al. (2019b) for counterexamples. However, for each cograph *G*, there exists a coloring $\sigma$ (with a sufficient number of colors) such that $(G,\sigma )$ is an *hc*-cograph.

### Proposition 3

(Thm. 9 in (Geiß et al. 2019b)) A graph $(G,\sigma )$ is a co-RBMG if and only if it is an *hc*-cograph.

Since orthology relations are necessarily cographs we can interpret Proposition 3 as necessary condition for an RBMG to correctly represent orthology.

The recursive construction of $(G,\sigma )$ in Def. 9 also defines a corresponding *hc*-cotree $(T^G_{hc },t_{hc },\sigma )$ whose leaves are the vertices of $(G,\sigma )$, i.e., the $(K_1,\sigma )$ appearing in (K1). Each internal node *u* of $T^G_{hc }$ corresponds to either a join (K2) or a disjoint union (K3) and is labeled by  such that  if *u* represents a join, and $t_{hc }(u)=\square$ if *u* corresponds to a disjoint union. Each inner vertex *u* of $T^G_{hc }$ represents the induced subgraph $(G,\sigma )[L(T^G_{hc }(u))]$.

### Proposition 4

(Thm. 10 in (Geiß et al. 2019b)) Every co-RBMG $(G,\sigma )$ is explained by its *hc*-cotree $(T^G_{hc },t_{hc },\sigma )$.

Now let $(T^G_{hc },t_{hc },\sigma )$ be the *hc*-cotree of a co-RBMG $(G,\sigma )$. Note, the structure of $T^G_{hc }$ is solely determined by the *hc*-cograph structure of $(G,\sigma )$. Somehwat surprisingly, the mathematical structure of the *hc*-cotree $(T^G_{hc },t_{hc },\sigma )$ and, in particular, its coloring $t_{hc }$ has a simple biological interpretation. Consider $\{v',v''\}=\mathsf {child}(u)$. If  in the *hc*-cotree, then $\sigma (L(T^G_{hc }(v')))\cap \sigma (L(T^G_{hc }(v'')))=\emptyset$ in agreement with Lemma 2. On the other hand, if $t_{hc }(u)=\square$, then (K3) implies $\sigma (L(T^G_{hc }(v')))\cap \sigma (L(T^G_{hc }(v'')))\ne \emptyset$, in which case *u* indeed must be a duplication from the biological point of view (contraposition of Lemma 2).

The *hc*-cotree $(T^G_{hc },t_{hc },\sigma )$ of $(G,\sigma )$ will in general not be discriminating and it is not necessarily possible to reduce $(T^G_{hc },t_hc ,\sigma )$ to a discriminating *hc*-cotree $({\hat{T}}^G_{hc },{\hat{t}},\sigma )$ that still explains $(G,\sigma )$. Although it is always possible to contract edges *uv* of $(T^G_{hc },t_{hc },\sigma )$ with  (cf. (Geiß et al. 2019b, Cor. 11)), there are examples where edges *uv* with $t_{hc }(u) = t_{hc }(u) =\square$ cannot be contracted to obtain a tree that still explains $(G,\sigma )$ (cf. (Geiß et al. 2019b, Fig. 15)). We refer to (Geiß et al. 2019b) for more details and a characterization of edges that are contractable. It is of interest, therefore, to ask whether there are true orthology relations $\varTheta$ that are not *hc*-cographs, or equivalently, when does a discriminating *hc*-cotree $({\hat{T}},{\hat{t}},\sigma )$ that is obtained by edge-contraction from a given *hc*-cotree $(T^G_{hc },t_{hc },\sigma )$ still explains an RBMG $(G,\sigma )$? To answer this question we provide first

### Definition 10

A tree $(T,t,\sigma )$ contains *no losses*, if for all $x\in V(T)$ with $t(x)=\square$ we have $\sigma (L(T(v'))) = \sigma (L(T(v'')))$ for all $v', v''\in \mathsf {child}(x)$.

### Theorem 4

Let $(T,\sigma )$ be a leaf-labeled tree such that there is a reconciliation map $\mu$ to some species tree and assume that $(T,t_{\mu },\sigma )$ does not contain losses. Then

1. The RBMG $G(T,\sigma )$ explained by $(T,\sigma )$ equals the colored cograph $(\varTheta (T,t_{\mu }),\sigma )$.
2. The unique disciminating cotree $({\hat{T}}, {\hat{t}}, \sigma )$ of $(\varTheta (T,t_{\mu }),\sigma )$ explains the RBMG $(G,\sigma )$.

### Proof

To simplify the notation, we set $(G,\sigma ) = G(T,\sigma )$ and $(H,\sigma )=(\varTheta (T,t_{\mu }),\sigma )$.

We start with proving Statement (1). By Theorem 2, $(H,\sigma )$ is a subgraph of $(G,\sigma )$ and $V(H)=V(G)$, hence it suffices to show that every edge $ab\in E(G)$ is also contained in *E*(*H*). Assume, for contradiction, that this is not the case, i.e., $ab \notin E(H)$, and thus $t_{\mu }(x)=\square$ for $x:={{\,\mathrm{lca}\,}}_T(a,b)$. Since $(T,t,\sigma )$ has no losses, we have $\sigma (L(T(v'))) = \sigma (L(T(v'')))$ for all $v', v''\in \mathsf {child}(x)$, and thus $a\in L(T(v'))$ and $b\in L(T(v''))$ for some pair of distinct children $v',v''\in \mathsf {child}(x)$ of *x*. From $\sigma (L(T(v'))) = \sigma (L(T(v'')))$ we know that there is a vertex $a'\in L(T(v''))$ with $\sigma (a')=\sigma (a)$. Thus, ${{\,\mathrm{lca}\,}}_T(a,b)=x\succ _T {{\,\mathrm{lca}\,}}_T(a',b)$ for some $a'\in L(T(v''))$, which implies that $ab\notin E(G)$; a contradiction. We conclude that $ab\in E(G)$ if and only if $ab\in E(H)$ and thus $(G,\sigma ) = (H,\sigma )$.

Let us now turn to Statement (2). In order to show that $({\hat{T}}, {\hat{t}}, \sigma )$ explains the RBMG $(G,\sigma )$ we first note that, since $(G,\sigma )$ is a cograph by Statement (1), there is a unique discriminating cotree $({\hat{T}}, {\hat{t}}, \sigma )$ for $(G,\sigma )$. Furthermore, $({\hat{T}}, {\hat{t}}, \sigma )$ is obtained from any cotree $(T,t_{\mu },\sigma )$ for $(G,\sigma )$ by contracting all edges *uv* in *T* with $t_{\mu }(u)=t_{\mu }(v)$ (Hellmuth et al. 2013). It remains to show that *ab* is an edge in $(G,\sigma )$ if and only if *ab* forms a reciprocal best match in $({\hat{T}}, \sigma )$.

First consider duplications. Suppose, we have contracted the edge *xv* with $t_{\mu }(x)=t_{\mu }(v) = \square$. By assumption, for all children $v',v''$ of *v* we have $\sigma (L(T(v'))) = \sigma (L(T(v'')))$. Moreover, since $\sigma (L(T(v)))$ is the union of species $\sigma (L(T(w))))$ of its children *w*, we have $\sigma (L(T(v))) = \sigma (L(T(v')))=\sigma (L(T(v'')))$. Hence, after contraction of *xv*, the vertices $v'$ and $v''$ are now children of *x* and still satisfy $\sigma (L({\hat{T}}(v'))) = \sigma (L({\hat{T}}(v'')))$. In particular, $\sigma (L({\hat{T}}(v'))) = \sigma (L({\hat{T}}(w)))$ for every child *w* of *x*. By induction on the number of contracted edges, every vertex *x* in ${\hat{T}}$ with ${\hat{t}}(x)=\square$ still satisfies $\sigma (L({\hat{T}}(v'))) = \sigma (L({\hat{T}}(v'')))$ for all children $v',v''$ of *x* in ${\hat{T}}$. Thus, the same argument as in the proof of Statement (1) implies that *ab* cannot be a reciprocal best match in ${\hat{T}}$ for all $a\in L(T(v'))$ and $b\in L(T(v''))$. We also have ${{\,\mathrm{lca}\,}}_{{\hat{T}}}(a,b)=x$ for $a\in L(T(v'))$ and $b\in L(T(v''))$, and thus ${\hat{t}}({{\,\mathrm{lca}\,}}_{{\hat{T}}}(a,b)) = \square$. Since $({\hat{T}}, {\hat{t}}, \sigma )$ is a cotree for the cograph $(G,\sigma )$, ${\hat{t}}({{\,\mathrm{lca}\,}}_{{\hat{T}}}(a,b)) = \square$ implies $ab\notin E(G)$. Therefore, $ab\notin E(G)$ unless *a* and *b* form a reciprocal best match in $({\hat{T}},\sigma )$.

Let us now turn to speciation vertices. Lemma 47 in (Geiß et al. 2019b) states, in particular, that all non-discriminating edges *uv* with  can be contracted to obtain a tree that still explains $(G,\sigma )$. Thus, if *a* and *b* are reciprocal best matches in $({\hat{T}},\sigma )$, then $ab\in E(G)$. We conclude, therefore, that $ab\in E(G)$ if and only if *a* and *b* are reciprocal best matches in $({\hat{T}},\sigma )$. $\square$

Prop. 3 shows that if the *no loss* condition of Def. 10 holds, then $(\varTheta (T,t_{\mu }),\sigma )=G(T,\sigma )$ is a co-RBMG, an *hc*-cograph, and an orthology relation.

The *no loss* condition of Def. 10 is very restrictive, however, and thus in general will not be satisfied in real-life data. Theorem 1 shows that orthology relations correspond to properly colored cographs with compatible sets of the informative triples. The characterization of co-RBMGs in (Geiß et al. 2019b), on the other hand, shows that only *hc*-colorings may appear. Since the requirement that $\sigma$ is a proper coloring already implies disjointness of the color sets for join operations, we can interpret the *hc*-coloring condition as a condition on duplication vertices. The offending vertices are exactly those for which (i) $t(u)=\square$ and (ii) there are two children $v',v''\in \mathsf {child}(u)$ such that both $\sigma (L(T(v')))\setminus \sigma (L(T(v'')))\ne \emptyset$ and $\sigma (L(T(v'')))\setminus \sigma (L(T(v')))\ne \emptyset$. In this case, there is a pair of species such that a different “paralog group” (that is, a lineage of genes descending from a duplication) is missing in each of them. Every pair of vertices $a\in L(T(v'))$ with $\sigma (a)\notin \sigma (L(T(v'')))$ and $b\in L(T(v''))$ with $\sigma (b)\notin \sigma (L(T(v')))$ forms a best match and thus a false positive orthology assignment. Since an RBMG is a cograph only if it is hierarchically colored, the presence of such duplications implies that the RBMG is not a cograph. At least in principle, therefore, it should be possible to identify the false positive edges by means of a suitable cograph-editing approach.

Before closing this section, we briefly return to the existence of reconciliation maps. Since every hc-cograph is a properly colored cograph, Theorem 1 immediately implies

### Corollary 4

Let $\varTheta$ be an *hc*-cograph with vertex set *L* and associated *hc*-cotree $(T^{\varTheta }_{hc },t_{hc },\sigma )$ with leaf set *L*. Then there exists a reconciliation map $\mu$ from $(T^{\varTheta }_{hc },t_{hc },\sigma )$ to some species tree *S* if and only if ${\mathcal {S}}(T_{\varTheta },t_{\varTheta },\sigma )$ is compatible.

By Cor. 4, it is not necessarily possible to reconcile a (discriminating) *hc*-cotree with any species tree. An example is shown in Fig. 5. To be more precise, the *hc*-cotree $(T^G_{hc },t_{hc },\sigma )$ in Fig. 5 yields the conflicting species triples *AB*|*C* and *AC*|*B*. Hence, Prop. 1 implies that $(T^G_{hc },t_{hc },\sigma )$ cannot be reconciled with any species tree even though $(T^G_{hc },\sigma )$ explains the RBMG $(G,\sigma )$. One can contract edges of $(T^G_hc ,\sigma )$ to obtain a least resolved tree $(T^*,\sigma )$ that still explains $(G,\sigma )$, see Fig. 5 (top right). In agreement with Lemma 7, ${\mathcal {S}}(T^*,t_{\mu },\sigma ) = \emptyset$ and thus, there is always a reconciliation map $\mu$ from $(T^*,t_{\mu },\sigma )$ to any species tree *S* with $L(S)=\sigma (L(T))$. Moreover, in agreement with Theorem 2, all orthologous pairs in $\varTheta (T^*,{\hat{t}_T},\sigma )$ are best matches. Although $(T^*,\sigma )$ explains $(G,\sigma )$, the two graphs $(G,\sigma ) = (\varTheta (T^G_hc ,t),\sigma )$ and $(\varTheta (T^*,{\hat{t}_T}),\sigma )$ are very different. In particular, by Corollary 3, $\varTheta (T^*,{\hat{t}_T})$ is the disjoint union of cliques.

### Observation 2

In general it is not necessary to edit $(G,\sigma )$ to a disjoint union of cliques to obtain a valid orthology relation.

An example is provided by the tree $(T,{\hat{t}_T},\sigma )$ in Fig. 5. Obviously, $\varTheta (T,{\hat{t}_T})$ is not the disjoint union of cliques. Moreover, *AB*|*C* is the only informative triple displayed by $(T,{\hat{t}_T},\sigma )$ where *A*, *B*, and *C* correspond to the red, blue and green species, respectively. Prop. 1 implies that $(T,{\hat{t}_T},\sigma )$ can be reconciled with any species tree that displays *AB*|*C*. In other words, $\varTheta (T,{\hat{t}_T})$ is already “biologically feasible” and there is no need to remove further edges from $\varTheta (T,{\hat{t}_T})$.

## Non-orthologous reciprocal best matches

In this section we investigate to what extent false positive orthology assignments in the reciprocal best match graph can be identified. Since the orthology relation $\varTheta$ must be a cograph, it is natural to consider the smallest obstructions, i.e., induced $P_4$s in more detail. First we note that every induced $P_4$ in an RBMG contains either three or four distinct colors (Geiß et al. 2019b, Sect. E). Each $P_4$ in an RBMG $(G,\sigma )$ spans an induced subgraph of every BMG $(\vec {G},\sigma )$ that contains $(G,\sigma )$ as its symmetric part. These induced subgraphs of a BMG $(\vec {G},\sigma )$ with four vertices are known as *quartets*. With respect to a fixed BMG, every induced $P_4$ belongs to one of three distinct types which are defined in terms of its coloring and the quartet in which it resides. An induced $P_4$ with edges *ab*, *bc*, and *cd* is denoted by $\langle abcd \rangle$ or, equivalently, $\langle dcba \rangle$.

### Definition 11

Let $(\vec {G},\sigma )$ be a BMG explained by the tree $(T,\sigma )$, with symmetric part $(G,\sigma )$ and let $Q:=\{x,x',y,z\} \subseteq L(T)$ with $\sigma (x)=\sigma (x')$ and pairwise distinct colors $\sigma (x)$, $\sigma (y)$, and $\sigma (z)$. The set *Q*, resp., the induced subgraph $(\vec {G}_{|Q},\sigma _{|Q})$ is

- a *good quartet* if (i) $\langle xyzx'\rangle$ is an induced $P_4$ in $(G,\sigma )$ and (ii) $(x,z),(x',y)\in E(\vec {G})$ and $(z,x),(y,x')\notin E(\vec {G})$,
- a *bad quartet* if (i) $\langle xyzx'\rangle$ is an induced $P_4$ in $(G,\sigma )$ and (ii) $(z,x),(y,x')\in E(\vec {G})$ and $(x,z),(x',y)\notin E(\vec {G})$, and
- an *ugly quartet* if $\langle xyx'z\rangle$ is an induced $P_4$ in $(G,\sigma )$.

**Fig. 6:**
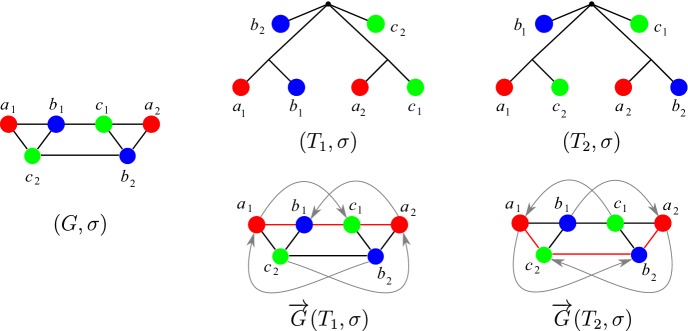
The 3-RBMG $(G,\sigma )$ is explained by two trees $(T_1,\sigma )$ and $(T_2,\sigma )$. These induce distinct BMGs $\vec {G}(T_1,\sigma )$ and $\vec {G}(T_2,\sigma )$. In $\vec {G}(T_1,\sigma )$, $P^1 =\langle a_1b_1c_1a_2\rangle$ defines a good quartet, while $P^2 =\langle a_1c_2b_2a_2\rangle$ induces a bad quartet. In $\vec {G}(T_2,\sigma )$ the situation is reversed. The good quartets in $\vec {G}(T_1,\sigma )$ and $\vec {G}(T_2,\sigma )$ are indicated by red edges. The induced paths $\langle a_1 b_1 c_1 b_2\rangle$ and $\langle a_2 c_1 b_1 c_2\rangle$ are examples of ugly quartets. Figure reused from (Geiß et al. 2019b), ©Springer (color figure online)

If *Q* is a good, bad, or ugly quartet we will refer to the underlying induced $P_4$ as a good, bad, or ugly quartet, respectively. Lemma 32 of (Geiß et al. 2019b) states that every quartet *Q* in an RBMG $(G,\sigma )$ that is contained in a BMG $(\vec {G},\sigma )$ is either good, bad, or ugly. An example of an RBMG containing good, bad, and ugly quartets is shown in Fig. 6. Note that good, bad, and ugly quartets cannot appear in RBMGs of Type (A). These are cographs and thus by definition do not contain induced $P_4\hbox {s}$.

The location of good quartets (in contrast to bad and ugly quartets) turns out to be strictly constrained. This fact can be used to show that the “middle” edge of any good quartet must be a false positive orthology assignment:

### Lemma 8

Let $(T,\sigma )$ be some leaf-labeled tree and ${\hat{t}}_T$ the extremal event labeling for $(T,\sigma )$. If $\langle xyzx'\rangle$ is a good quartet in the BMG $\vec {G}(T,\sigma )$, then ${\hat{t}_T}(v)=\square$ for $v:={{\,\mathrm{lca}\,}}(x,x',y,z)$.

### Proof

Lemma 36 of Geiß et al. (2019b) implies that for a good quartet $\langle xyzx' \rangle$ in $\vec {G}(T,\sigma )$ with $v:={{\,\mathrm{lca}\,}}(x,x',y,z)$ there are two distinct children $v_1, v_2\in \mathsf {child}(v)$ such that $x,y \preceq _T v_1$ and $x',z\preceq _T v_2$. Thus, in particular, $v_1$ and $v_2$ must be inner vertices in $(T,\sigma )$. Since $\sigma (x)=\sigma (x')$ by definition of a good quartet, we have $\sigma (L(T(v_1)))\cap \sigma (L(T(v_2)))\ne \emptyset$. Hence,  by definition of ${\hat{t}_T}$ (cf. Definition 6). $\square$

As an immediate consequence of Lemma 8 and Cor. 1, an analogous statement is true for event labelings $t_\mu$ for a given reconciliation map:

### Corollary 5

Let *T* and *S* be planted trees, $\sigma : L(T)\rightarrow L(S)$ a surjective map, and $\mu$ a reconciliation map from $(T,\sigma )$ to *S*. If $\langle xyzx'\rangle$ is a good quartet in the BMG $\vec {G}(T,\sigma )$, then $t_\mu (v)=\square$ for $v:={{\,\mathrm{lca}\,}}(x,x',y,z)$.

Given an RBMG $(G,\sigma )$ that contains a good quartet $\langle xyzx' \rangle$ (w.r.t. to the underlying BMG $(\vec {G},\sigma )$), the edge *yz* therefore always corresponds to a false positive orthology assignment, i.e., it is not contained in the true orthology relation $\varTheta$.

**Fig. 7:**
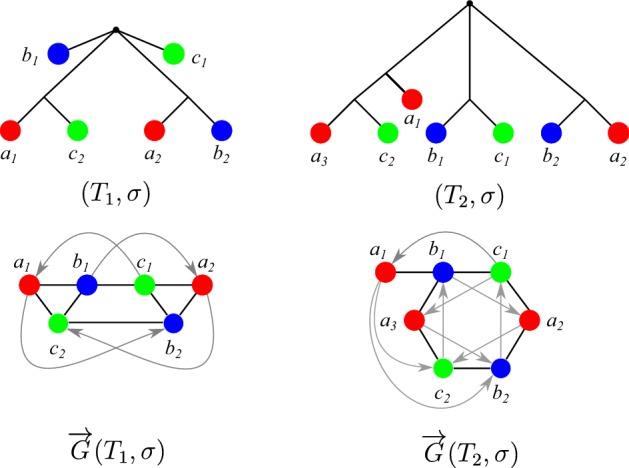
Not all false positive orthology assignments can be identified using good quartets. Conversely, bad and ugly quartets do not unambiguously identifiy false positive edges. See the text below Cor. 5 for explanation (color figure online)

Not all false positives can be identified in this way from good quartets, however. The RBMG $G(T_1,\sigma )$ in Fig. 7, for instance, contains only one good quartet, that is $\langle a_1c_2b_2a_2 \rangle$. After removal of the false positive edge $c_2b_2$, the remaining undirected graph still contains the bad quartet $\langle a_1b_1c_1a_2 \rangle$, hence, in particular, it still contains an induced $P_4$ and is, therefore, not an orthology relation.

Neither bad nor ugly quartets can be used to unambiguously identify false positive edges. For an example, consider Fig. 7. The two 3-RBMGs $G(T_1,\sigma )$ and $G(T_2,\sigma )$ both contain the bad quartet $\langle a_1 b_1 c_1 a_2 \rangle$. As a consequence of Lemma 2, neither the root of $T_1$ nor the root of $T_2$ can be labeled by a speciation event. Hence, as $a_1,b_1,c_1,a_2$ reside all in different subtrees below the root of $T_1$, all edges $a_1b_1, b_1c_1, c_1a_2$ in $G(T_1,\sigma )$ correspond to false positive orthology assignments. On the other hand, the vertices $b_1$ and $c_1$ reside within the same 2-colored subtree below the root of $T_2$ and are incident to the same parent in $T_2$. Therefore, one easily checks that there exist reconciliation scenarios where $b_1$ and $c_1$ are orthologous, hence the edge $b_1c_1$ must indeed be contained in the orthology relation. Similarly, $\langle a_1 b_1c_1b_2 \rangle$ and $\langle a_1 b_1a_3c_2 \rangle$ are ugly quartets in $G(T_1,\sigma )$ and $G(T_2,\sigma )$, respectively. By the same argumentation as before, the edges $a_1b_1$, $b_1c_1$, and $c_1b_2$ are false positives in $G(T_1,\sigma )$. For $(T_2,\sigma )$, however, there exist reconciliation scenarios, where $a_3$ and $c_2$ are orthologs.

Cor. 9 of Geiß et al. (2019b), finally, implies that every (B)-RBMG and every (C)-RBMG contains at least one good quartet. In particular, therefore, there is at least one false positive orthology assignment that can be identified with the help of good quartets. We shall see in Sect. 7.2, using simulated data, that in practice the overwhelming majority of false positive orthology assignments is already identified by good quartets.

From a theoretical point of view it is interesting nevertheless that it is possible to identify even more false positive orthology assignments starting from Lemma 2. It implies that $t({{\,\mathrm{lca}\,}}(x,y))=\square$ whenever *x* and *y* are located in two distinct leaf sets defined for the the same connected component of an induced 3-RBMG of Type (B) or (C). Details can be found in (Geiß et al. 2019b, Lemma 25) and the Supplemental Material. At least in our simulation data scenarios of this type that are not covered already by a good quartet seem to be exceedingly rare, and hence of little practical relevance.

## Simulations

Although the edges in the RMBG cannot identify orthologous pairs with certainty (as a consequence to Lemma 3), there is a close resemblance in practice, i.e., for empirically determined scenarios. In order to explore this connection in more detail, we consider simulated evolutionary scenarios $(T,S,\mu )$. These uniquely determine both the (reciprocal) best match graph $\vec {G}(T,\sigma )$ and $G(T,\sigma )$, resp., and the orthology graph $\varTheta$, thus allowing a direct comparison of these graphs. Since we only analyze scenarios $(T,S,\mu )$, we did not use simulations tools such as ALF (Dalquén et al. 2011) that are designed to simulate sequence data.

### Simulation methods

In order to simulate evolutionary scenarios $(T,S,\mu )$ we employ a stepwise procedure:

1. **Construction of the species tree***S*. We regard *S* as an ultrametric tree, i.e., its branch lengths are interpreted as real-time. Given a user-defined number of species *N* we generate *S* under the *innovations model* as described by Keller-Schmidt and Klemm (2012). The binary trees generated by this model have similar depth and imbalances as those of real phylogenetic trees from databases.
2. **Construction of the true gene tree**${\tilde{T}}$. Traversing the species tree *S* top-down, one gene tree ${\tilde{T}}$ is generated with user-defined rates $r_D$ for duplications, $r_L$ for losses, and $r_H$ for horizontal transfer events. The number of events along each edge of the species tree, of each type of event, is drawn from a Poisson distribution with parameter $\lambda = \ell r_e$, where $\ell$ is the length of the edge *e* and $r_e$ is the rate of the event type. Duplication and horizontal transfer events duplicate an active lineage and occur only inside edges of *S*. For duplications, both offspring lineages remain inside the same edge of the species tree as the parental gene. In contrast, one of the two offsprings of an HGT event is transferred to another, randomly selected, branch of the species tree at the same time. At speciation nodes all branches of the gene tree are copied into each offspring. Loss events terminate branches of $\tilde{T}$. Loss events may occur only within edges of the species tree that harbor more than one branch of the gene tree. Thus every leaf of *S* is reached by at least one branch of the gene tree ${\tilde{T}}$. All vertices *v* of ${\tilde{T}}$ are labeled with their event type *t*(*v*), in particular, there are different leaf labels for extant genes and lost genes. The simulation explicitly records the reconciliation map, i.e., the assignment of each vertex of ${\tilde{T}}$ to a vertex or edge of *S*.
3. **Construction of the observable gene tree***T***from**${\tilde{T}}$. The leaves of ${\tilde{T}}$ are either observable extant genes or unobservable losses. As described by Hernandez-Rosales et al. (2012), we prune ${\tilde{T}}$ in bottom-up order by removing all loss events and omitting all inner vertices with only a single remaining child.

Using steps (1) and (2), we simulated 10,000 scenarios for species trees with 3 to 100 species (=leaves) and additional 4000 scenarios for species trees with 3 to 50 leaves, drawn from a uniform distribution. For each of these species trees, exactly one gene tree was simulated as described above. The rate parameters were varied between 0.65 and 0.99 in steps of 0.01 for duplication and loss events. For HGTs, a rate in the range between 0.1 and 0.24, again in steps of 0.01, was used. A detailed list of all simulated scenarios can be found in the Supplemental Material. For each of the 14,000 true gene trees ${\tilde{T}}$ the total number $S_n$ of speciation events, $L_n$ of losses, $D_n$ of duplications, and $H_n$ of HGTs was determined. Summary statistics of the simulated scenarios are compiled in the Supplemental Material.

From each true gene tree ${\tilde{T}}$ we extracted the observable gene tree *T* as described in Step (3). For all retained vertices the reconciliation map $\mu$ and thus the event labeling $t=t_{\mu }$ remains unchanged. Since ${{\,\mathrm{lca}\,}}_T(x,y)={{\,\mathrm{lca}\,}}_{{\tilde{T}}}(x,y)$ for all extant genes $x,y\in L(T)$, it suffices to consider *T*. The leaf coloring map $\sigma :L(T)\rightarrow L(S)$ is obtained from its definition, i.e., setting $\sigma (v)=\mu (v)$ for all $v\in L(T)$. We can now extract the orthology relation and reciprocal best match relation from each scenario.

The orthology relation $\varTheta (T,t)$ is easily constructed from the event labeled gene tree (*T*, *t*), since $xy \in \varTheta (T,t)$ if and only if . An efficient way to compute $\varTheta (T,t)$ and the RBMG $(G,\sigma )$ that avoids the explicit evaluation of ${{\,\mathrm{lca}\,}}_T()$ is described in the Supplemental Material. For each reconciliation scenario $(T,S,\mu )$, we also identify all good quartets in the BMG $(\vec {G},\sigma )$ and then delete the middle edge of the corresponding $P_4$ from the RBMG $(G,\sigma )$. The resulting graph will be referred to as $(G_4,\sigma _4)$.

### Simulation results for duplication/loss scenarios

In order to assess the practical relevance of co-RBMGs we measured the abundance of non-cograph components in the simulated RBMGs. More precisely, we determined for each simulated RBMG the connected components of its restrictions to any three distinct colors and determined whether these components are cographs, graphs of Type (B), or graphs of Type (C). In order to identify these graph types, we used algorithms of (Hoàng et al. 2013) to first identify an induced $P_4$ belonging to a good quartet. If one exists, we check for the existence of an induced $P_5$ and then test whether its endpoints are connected, thus forming a hexagon characteristic for the a Type (C) graph. Otherwise, the presence of the $P_4$ implies Type (B), while the absence of induced $P_4$s guarantees that the component is a cograph.

We did not encounter a single Type (C) component in 14,000 simulated scenarios. As we shall see this is a consequence of the fact that all simulated trees are binary. To see this, we consider the structure of connected 3-RBMG of Type (C) in some more detail, generalizing some technical results by Geiß et al. (2019b):

#### Lemma 9

Let $$(G,\sigma )$$ be a connected 3-RBMG containing the induced $$C_6$$$$\langle x_1 y_1 z_1 x_2 y_2 z_2\rangle $$ with three distinct colors *r*, *s*, and *t* such that $$\sigma (x_1)=\sigma (x_2)=r$$, $$\sigma (y_1)=\sigma (y_2)=s$$, and $$\sigma (y_1)=\sigma (y_2)=t$$. Then, every tree $$(T,\sigma )$$ that explains $$(G,\sigma )$$ must satisfy the following property: There exist distinct $$v_1,v_2,v_3\in \mathsf {child}(v)$$ where $$v:={{\,\mathrm{lca}\,}}_T(x_1,x_2,y_1,y_2,z_1,z_2)$$ such that either $$x_1,y_1\preceq _T v_1$$, $$x_2,z_1\preceq _T v_2$$, $$y_2,z_2\preceq _T v_3$$ or $$y_1,z_1\preceq _T v_1$$, $$x_2,y_2\preceq _T v_2$$, $$x_1,z_2\preceq _T v_3$$.

#### Proof

If $|V(G)|>6$, then, due to the connectedness of $\vec {G}$, at least one of the six vertices of the induced $C_6$ is adjacent to more than one vertex of one of the colors *r*, *s*, *t*, hence the first statement immediately follows from Lemma 39(iii) in Geiß et al. (2019b). Now consider the special case $|V(G)|=6$. By Cor. 9 of Geiß et al. (2019b), $\vec {G}(T,\sigma )$ contains a good quartet. W.l.o.g. let $\langle x_1 y_1 z_1 x_2\rangle$ be a good quartet, thus $(x_1,z_1),(x_2,y_1)\in E(\vec {G})$ and $(z_1,x_1),(y_1,x_2)\notin E(\vec {G})$. This, in particular, implies ${{\,\mathrm{lca}\,}}_T(x_2,z_1)\prec _T{{\,\mathrm{lca}\,}}_T(x_1,z_1)$, thus there are distinct children $v_1, v_2\in \mathsf {child}(v)$ such that $x_1\preceq _T v_1$ and $x_2, z_1\preceq _T v_2$. Moreover, as $x_1y_1\in E(G)$ and $(y_1,x_2)\notin E(\vec {G})$, we have ${{\,\mathrm{lca}\,}}_T(x_1,y_1)\prec _T {{\,\mathrm{lca}\,}}_T(x_2,y_1)$, hence $y_1\preceq _T v_1$. Now consider $y_2$. Since $x_1y_2\notin E(G)$ and $x_2y_2\in E(G)$, it must hold ${{\,\mathrm{lca}\,}}_T(x_2,y_2)\preceq _T {{\,\mathrm{lca}\,}}_T(x_1,y_2)$, hence $y_2\notin L(T(v_1))$. Assume, for contradiction, that $y_2\preceq _T v_2$. Then, as $y_2z_2\in E(G)$ and ${{\,\mathrm{lca}\,}}_T(y_2,z_1)\preceq _T v_2$, we clearly have $z_2\preceq _T v_2$. However, this implies ${{\,\mathrm{lca}\,}}_T(x_2,z_2)\prec _T{{\,\mathrm{lca}\,}}_T(x_1,z_2)$, contradicting $x_1z_2\in E(G)$. We therefore conclude that there must exist a vertex $v_3\in \mathsf {child}(v)\setminus \{v_1,v_2\}$ such that $y_2\preceq _T v_3$. One easily checks that this implies $z_2\preceq _T v_3$, which completes the proof. $\square$

#### Theorem 5

If $(T,\sigma )$ is a binary leaf-labeled tree, then $G(T,\sigma )$ does not contain a connected component of Type (C).

#### Proof

By Obs. 6 of (Geiß et al. 2019b), the restriction $(T_{rst},\sigma _{rst})$ of $(T,\sigma )$ explains the subgraph $(G_{rst},\sigma _{rst})$ of $G(T,\sigma )$ that is induced by vertices with color *r*, *s*, or *t*. Thm. 2 of (Geiß et al. 2019b) shows, furthermore, that every connected component of $(G_{rst},\sigma _{rst})$ is explained by restriction $(T',\sigma ')$ of $(T_{rst},\sigma _{rst})$ to the corresponding vertices. Now suppose $(T,\sigma )$ is a binary. Then both $(T_{rst},\sigma _{rst})$ and $(T',\sigma ')$ are also binary. By contraposition of Lemma 9, no $C_6$ as specified in Lemma 9 can be explained by $(T',\sigma ')$, and thus $G(T,\sigma )$ cannot contain a connected component of Type (C). $\square$

**Fig. 8:**
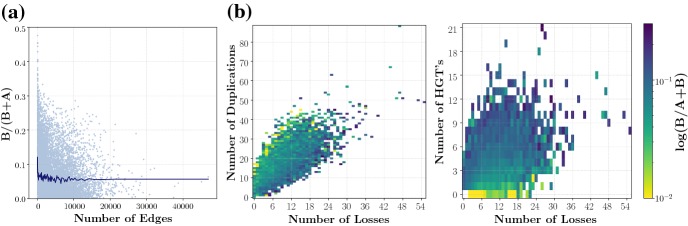
Relative abundance $\eta =\frac{B}{B+A}$ of (B)-RBMGs in the simulation data. Panel **a** shows the dependence on the number of edges in the BMG in every simulated scenario, and its average depicted by the line in darker blue. Scatter plots **b** show the dependence of $\eta$ on the number of duplications and losses, and HGTs and losses, respectively (color figure online)

Although events that generate more than two offspring lineages are logically possible in real data, most multifurcations in phylogenetic trees are considered to be “soft polytomies”, arising from data that are insufficient to produce a fully resolved, binary trees (Purvis and Garland Jr. 1993; Kuhn et al. 2011; Sayyari and Mirarab 2018). Type (C) 3-RBMGs thus should be very unlikely under biologically plausible assumptions on the model of evolution. Here we only consider the abundance of Type (B) components relative to all Type (A) and (B) components. We denote their ratio by $\eta$. The results are summarized in Fig. 8. We find that $\eta$ is usually below 20% and increases with the number of loss and HGT events. More precisely, 83.47% of the 14,000 scenarios have at least one Type (B) component and 16.53% do not have Type (B) components at all. Among all 3-colored connected components taken from the restrictions to any three colors, 94.41% are of Type (A) and 5.59% are of Type (B).

**Fig. 9:**
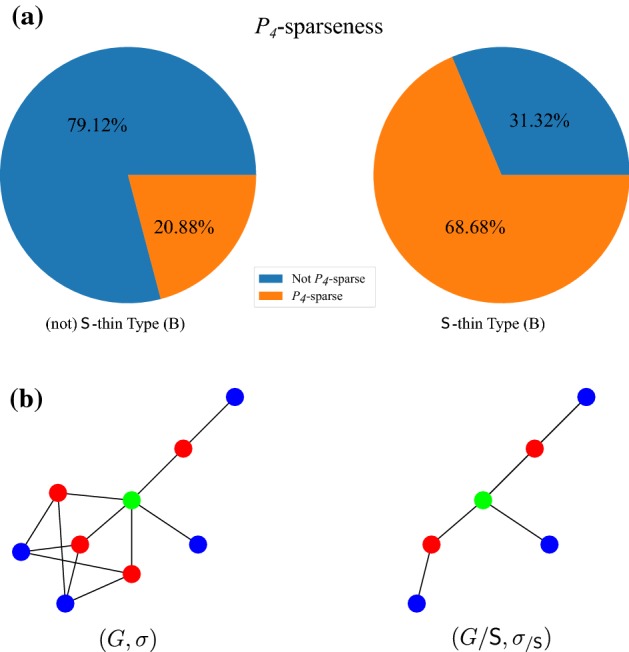
*Top:* Among our 14,000 simulated scenarios we found that a majority of 79.12% of the (not necessarily $\mathsf {S}$-thin) 3-colored Type (B) components are not $P_4$-sparse. For the corresponding $\mathsf {S}$-version of those 3-colored components only 31.32% are not $P_4$-sparse while 68.68% are $P_4$-sparse. *Below:* One of the simulated 3-colored Type (B) components $(G,\sigma )$, which is not $\mathsf {S}$-thin, and its corresponding $\mathsf {S}$-thin version $(G/\mathsf {S}, \sigma _{/\mathsf {S}})$ (color figure online)

A graph *G* is called $P_4$-sparse if every induced subgraph on five vertices contains at most one induced $P_4$ (Jamison and Olariu 1992). The interest in $P_4$-sparse graphs derives from the fact that the cograph editing problem is solvable in linear time from $P_4$-sparse graphs (Liu et al. 2012). It is of immediate practical interest, therefore, to determine the abundance of $P_4$-sparse RBMGs that are not cographs. Among the 14,000 simulated scenarios, we found that about 20.9% of the 3-colored Type (B) components are $P_4$-sparse, while the majority contains “overlapping” $P_4$s. We then investigated the corresponding $\mathsf {S}$-thin graphs. An undirected colored graph $(G,\sigma )$ is called $\mathsf {S}$-thin if no distinct vertices are in relation $\mathsf {S}$. Two vertices *a* and *b* are in relation $\mathsf {S}$ if $N(a)=N(b)$ and $\sigma (a)=\sigma (b)$. Somewhat surprisingly, this yields a reversed situation, where more than two thirds of the $\mathsf {S}$-thin 3-colored Type (B) components are now $P_4$-sparse, while only a minority of 31.32% is not $P_4$-sparse. An example of an undirected colored graph $(G,\sigma )$ and its corresponding $\mathsf {S}$-thin version $(G/\mathsf {S}, \sigma _{/\mathsf {S}})$, which we found during our simluations, is shown in Panel (B) of Fig. 9.

**Fig. 10:**
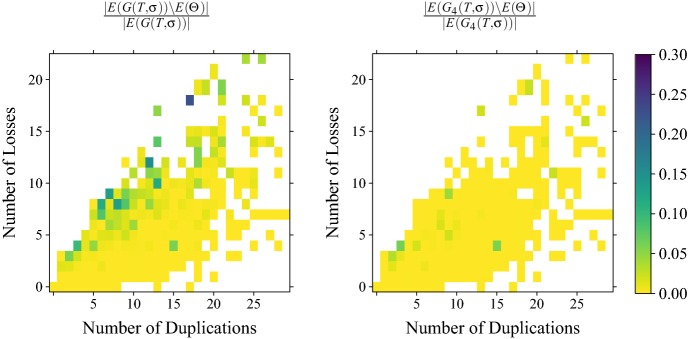
Fraction of non-orthology edges in the reciprocal best match graph (l.h.s.). The *x*-axis, resp., *y*-axis indicate the total number of duplications, resp., losses in the simulated scenarios. Most of the false positive orthology assignments in the l.h.s. panel are removed by deleting the middle edge of good quartets (r.h.s. panel). White background indicates *no data* (color figure online)

Next we investigated the relationship of the RBMG $G(T,\sigma )$ and the orthology graph $\varTheta$ (see Fig. 10). We empirically confirmed that $E(\varTheta )\subseteq E(G(T,\sigma ))$ in the absence of HGT (not shown). Also following our expectations, the fraction $|E(G(T,\sigma ))\setminus E(\varTheta )|/|E(G(T,\sigma ))|$ of false-positive orthology predictions in an RBMG is small as long as duplications and losses remain moderate (l.h.s. panel in Fig. 10). Most of the false positive orthology calls are associated with large numbers of losses for a given number of duplications.

We find that good quartets eliminate nearly all false positive edges from the RBMG and leave a nearly perfect orthology graph (r.h.s. panel in Fig. 10). As we have seen so far, reciprocal best matches indeed form an excellent approximation of orthology in duplication-loss scenarios. In particular, the good quartets identify nearly all false positive edges, making it easy to remove the few remaining $P_4\hbox {s}$ using a generic cograph editing algorithm (Liu et al. 2012).

## Outlook: evolutionary scenarios with horizontal gene transfer

The benign results above beg the question how robust they are under HGT. Gene family histories with HGT have been a topic of intense study in recent years (Doyon et al. 2010; Tofigh et al. 2011; Bansal et al. 2012; Nøjgaard et al. 2018). Following the so-called DTL-scenarios as proposed e.g. by Tofigh et al. (2011), Bansal et al. (2012) we relax the notion of reconciliation maps, since ancestry is no longer preserved. We replace Axiom (R2) by

(R2w) *Weak Ancestor Preservation.*
  If $x\prec _T y$, then either $\mu (x)\preceq _S\mu (y)$ or $\mu (x)$ and $\mu (y)$ are incomparable w.r.t. $\prec _S$.

and add the following constraints

(R3.iii) *Addition to the Speciation Constraint.*
  If $\mu (x)\in W^0$, then $\mu (v)\preceq _T\mu (x)$ for all $v\in \mathsf {child}(x)$.
(R4) *HGT Constraint.*
  If *x* has a child *y* such that $\mu (x)$ and $\mu (y)$ are incomparable, then *x* also has a child $y'$ with $\mu (y')\preceq _S \mu (x)$.

Property (R2w) equivalently states that if $x\prec _T y$, then we must not have $\mu (y)\prec _S\mu (x)$, which would invert the temporal order. Property (R3.iii) (which follows from (R2) but not from (R2w)) ensures that the children of speciation events are still mapped to positions that are comparable to the image of the speciation node. Condition (R4), finally, requires that every horizontal transfer event also has a vertically inherited offspring. Note that condition (R4) is void if (R2) holds. In summary the axioms (R0), (R1), (R2w), (R3.i), (R3.ii), (R3.iii), and (R4) are a proper generalization of Def. 3. We note that these axioms are not sufficient to ensure time consistency, however. We refer to Nøjgaard et al. (2018) for details. Our choice of axioms also rules out some scenarios that may appear in reality (or simulations), but which are not observable when only evolutionary divergence is available as measurement. For example, Condition (R3.ii) excludes scenarios in which HGT events have no surviving vertically inherited offspring.

We furthermore extend the event map *t* for a gene tree *T* to include HGT as an additional event type denoted by the symbol $\triangle$. We define  such that $t(u)=\triangle$ if and only if *u* has a child *v* such that $\mu (u)$ and $\mu (v)$ are incomparable. Since the offsprings of an HGT event are not equivalent, it is useful to introduce an edge labeling $\lambda :E(T)\rightarrow \{0,1\}$ such that $\lambda (uv)=1$ if $\mu (u)$ and $\mu (v)$ are incomparable w.r.t. $\prec _S$. This edge labeling is investigated in detail by Geiß et al. (2018) as the basis of Fitch’s xenology relation. Alternatively, the asymmetry can be handled by enforcing an ordering of the vertices, see (Hellmuth et al. 2017).

**Fig. 11:**
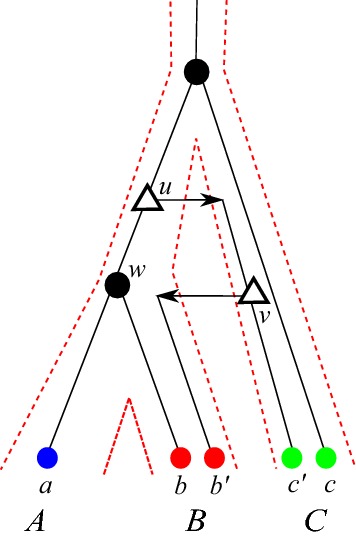
A gene tree $(T,t,\lambda ,\sigma )$ reconciled with a species tree *S*. Here, we have two transfer edges *uv* and $vb'$ with $t(u)=t(v)=\triangle$. For the two children *w* and *v* of *u* it holds $\sigma (L(T(w)))\cap \sigma (L(T(v)))\ne \emptyset$, a property that is shared with duplication vertices. For the two children $b'$ and $c'$ of *v* it holds $\sigma (L(T(b')))\cap \sigma (L(T(c')))= \emptyset$, a property that is shared with speciation vertices. In this example, *c* and $c'$ are xeno-orthologs and the pairs $(c,c'),(c',c)$ will be excluded from the resulting orthology relation (color figure online)

Evolutionary scenarios with horizontal transfer may lead to a situation where two genes *x*, *y* in the same species, i.e., with $\sigma (x)=\sigma (y)$, derive from a speciation, i.e., . This is the case when the two lineages underwent an HGT event that transferred a copy back into the lineage in which the other gene has been vertically transmitted. We call such genes *xeno-orthologs* and exclude them from the orthology relation, see Fig. 11. This choice is motivated (1) by the fact that, by definition, genes of the same species cannot be recognized as reciprocal best matches, and (2) from a biological perspective they behave rather like paralogs. In scenarios with HGT we therefore modify the definition of the orthology graph such that $E(G_1 {{\,\mathrm{\bowtie }\,}}G_2)$ is replaced by

3 $$\begin{aligned} E(G_1 {\tilde{{{\,\mathrm{\bowtie }\,}}}} G_2) := E(G_1) \cup E(G_2) \cup \{ uv \mid u \in V(G_1),v \in V(G_2) \text { and } \sigma (u) \ne \sigma (v) \}\,. \end{aligned}$$

The extremal map ${\hat{t}_T}$ as in Def. 6 cannot easily be extended to include HGT, as the events  and $\square$ on some vertex *u* are solely defined on two exclusive cases: either $\sigma (L(T(u_1)))$ and $\sigma (L(T(u_2)))$ are disjoint or not for $u_1,u_2\in \mathsf {child}(u)$. Both cases, however, can also appear when we have HGT (see Fig. 11 for an example). That is, the fact that $\sigma (L(T(u_1)))$ and $\sigma (L(T(u_2)))$ are disjoint or not, does not help to unambiguously identify the event types in the presence of HGT.

Prop. 1 can be generalized to the case that $(T,t,\lambda ,\sigma )$ contains HGT events. The existence of reconciliation maps from an event-labeled tree $(T,t,\lambda ,\sigma )$ to an *unknown* species tree can be characterized in terms of species triples $\sigma (a)\sigma (b)|\sigma (c)$ that can be derived from $(T,t,\lambda ,\sigma )$ as follows: Denote by $\mathcal {E}:=\{e\in E(T,t,\lambda ,\sigma )\mid \lambda (e)=1\}$ the set of all transfer edges in the labeled gene tree and let $(T_{\mathcal {\overline{E}}},t,\sigma )$ be the forest obtained from $(T,t,\lambda ,\sigma )$ by removing all transfer edges. By definition, $\mu (x)$ and $\mu (y)$ are incomparable for every transfer edge *xy* in *T*. The set ${\mathcal {S}}(T,t,\lambda ,\sigma )$ is the set of triples $\sigma (a)\sigma (b)|\sigma (c)$ where $\sigma (a)$, $\sigma (b)$, $\sigma (c)$ are pairwise distinct and either

1. *ab*|*c* is a triple displayed by a connected component $T'$ of $T_{\mathcal {\overline{E}}}$ such that the root of the triple is a speciation event, i.e., .
2. or $a,b\in L(T_{\mathcal {\overline{E}}}(x))$ and $c\in L(T_{\mathcal {\overline{E}}}(y))$ for some transfer edge *xy* or *yx* of *T*.

### Proposition 5

(Hellmuth 2017) Given an event-labeled, leaf-labeled tree $(T,t,\sigma )$. Then, there is a reconciliation map $\mu :V(T)\rightarrow V(S)\cup E(S)$ to some species tree *S* if and only if ${\mathcal {S}}(T,t,\sigma )$ is compatible. In this case, $(T,t,\sigma )$ can be reconciled with every species tree *S* that displays the triples in ${\mathcal {S}}(T,t,\sigma )$.

Here, we have not added additional constraints on reconciliation maps that ensure that the map is also “time-consistent”, that is, genes do not travel “back” in the species tree, see (Nøjgaard et al. 2018) for further discussion on this. However, Prop. 5 gives at least a necessary condition for the existence of time-consistent reconciliation maps. A simple proof of Prop. 5 for the case that *T* is binary and does not contain HGT events can be found in (Hernandez-Rosales et al. 2012). Moreover, generalizations of reconciling event-labeled gene trees with species networks have been established by Hellmuth et al. (2019).

**Fig. 12:**
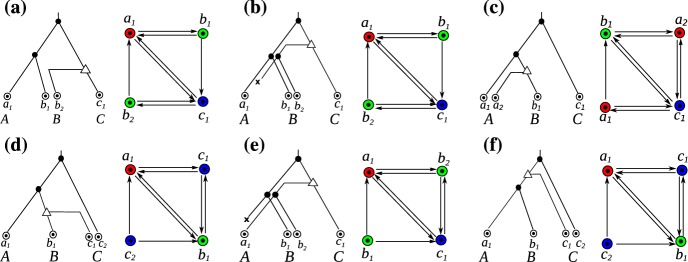
Scenarios with four genes, three species, and a single HGT event for which RBMG $G(T,\sigma )$ and orthology relation $\varTheta (T,t)$ differ. The BMG is shown for each scenario. In the first two cases (**a**) and (**b**), $G(T,\sigma )$ contains an induced $P_4$ in the RBMG, which might serve as indication for HGT events. In the remaining cases, the $G(T,\sigma )$ is a cograph, which does not represent the correct orthology relation, however. In scenario (**c**), the graph $G(T,\sigma )$ is a triangle with an attached edge, while the orthology relation is given by $\varTheta (T,t)=K_4-e-f$ with the missing edges $e=a_1a_2$ and $f=a_1b_1$, where the latter results from the xenologous pair $a_2,b_1$. In the remaining three cases (**d**)–(**f**), the RBMG is  compared to the orthology relation $\varTheta (T,t)=K_4-e$, where the edge *e* again corresponds to the edge between genes of the same species (color figure online)

**Fig. 13:**
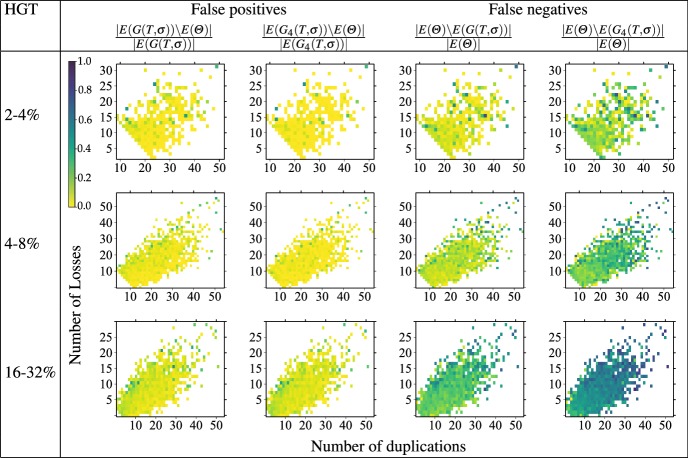
Dependence of the fraction of false positive and false negative orthology assignments in RBMGs in the presence of different levels of HGT, measured as percentage of HGT events among all events in the simulated true gene trees ${\tilde{T}}$. As in Fig. 10, data are shown as functions of the number of duplication and loss events in the scenario. While the number of false positives seems to depend very little on even high levels of HGT, the fraction of false negatives is rapidly increasing. Since HGT introduces good quartets that comprise only true orthology edges, their removal further increases the false positive rate (last column) (color figure online)

In contrast to pure DL scenarios, it is no longer guaranteed that all true orthology relationships are also reciprocal best matches. Figure 12 gives counterexamples. In three of these scenarios the RBMG contains an induced $P_4$ that mimics a good quartet. Removal of the middle edge of good quartets therefore not only reduces false positives in DL scenarios but also introduces additional false negatives in the presence of HGT (Fig. 13).

## Discussion

In the theoretical part of this contribution we have clarified the relationships between (reciprocal) best match graphs (RBMGs), orthology, reconciliation map, gene tree, species tree, and event map for the case of duplication loss scenarios.

The orthology graph $\varTheta$ is necessarily a subgraph of the RBMG. In the absence of HGT, RBMGs therefore produce only false positive but no false negative orthology assignments. Using not only reciprocal best matches but all best matches, furthermore, shows that good quartets identify almost all false positive edges. Removing the central edge of all good quartets in $(\vec {G},\sigma )$ yields nearly perfect orthology estimates. This, however, implies that orthology inference is not solely based on reciprocal best matches. Instead, it is necessary to also include certain directional best matches, namely those that identify good quartets.

We observed that a small number of HGT events can cause large deviations between the RBMG $(G,\sigma )$ and the orthology graph $\varTheta$. However, we have considered here the worst-case scenario, where HGT events occur between relatively closely related organisms. While this is of utmost relevance in some cases, for instance for toxin and virulence genes in bacteria, it is of little concern e.g. for the evolution of animals. In the latter case, xenologs almost always originate from bacteria or viruses, i.e., from outgroups. The xenologs then form their own group of co-orthologs and behave as if they would have been lost in the species outside the subtree that received the horizontally transfered gene.

From a more theoretical point of view, our empirical findings in the HGT case beg two questions: (1) Are there *local* features in the (R)BMG that make it possible to unambiguously identify HGT, at least in some cases? (2) What kind of additional information can be integrated to distinguish good quartets arising from duplication/loss events that can be safely removed from those that are introduced by HGT and should be “repaired” in a different manner. Most obviously, one may ask whether the Fitch relation is sufficient (we conjecture that this is the case) (Geiß et al. 2018; Hellmuth and Seemann 2019), or whether it suffices to know that a leaf is a (recent) result of transfer (we conjecture that this is not enough in general).

The identification of edges in the RBMG that should or should not be removed has important implication for orthology detection approaches that enforce the cograph structure of the predicted orthology relation by means of cograph editing. While this is an NP-complete problem (Liu et al. 2012) in general, the complexity of the colored version, i.e., editing a properly colored graph to the nearest *hc*-cograph remains open. The removal of false positive edges identified by good quartets empirically reduces the number of induced $P_4\hbox {s}$ drastically. This observation also suggests to consider *hc*-cograph editing with a given best match relation. We suspect that the additional knowledge of the directed edges makes the problem tractable since it already implies a unique least resolved tree that captures much of the cograph structure.

Cograph editing would be fully content with *hc*-cographs, i.e., co-RBMGs. These are not necessarily “biologically feasible” in the sense that they can be reconciled with a species tree. It will therefore be of interest to consider the problem of editing an *hc*-cograph to another *hc*-cograph that is reconcilable with some or a given species tree – a problem that has been considered already for orthology relations (Lafond et al. 2016; Lafond and El-Mabrouk 2014). Since the obstructions are conflicting triples with a speciation at their top node, the offending data are conflicting orthology assignments. It seems natural therefore to phrase the problem not as an arbitrary editing problem but instead to ask for a maximal induced sub-*hc*-cograph that implies a compatible triple set. If it is indeed true that triples necessarily displayed by the species tree can be extracted directly from the c(R)BMG, it will be of practical use to consider the corresponding edge deletion problem for c(R)BMGs. In particular, it would be interesting to know whether the latter problem is the same as asking for the maximal compatible subset of triples implied by the c(R)BMG or co-BMG?

## Electronic supplementary material

Below is the link to the electronic supplementary material.

Supplementary material 1 (pdf 215 KB)
