# Supplementary material for: Best match graphs and reconciliation of gene trees with species trees
Source: J Math Biol. 2020 Jan 30;80(5):1459–95. doi: 10.1007/s00285-020-01469-y (PMC7052050; doi:10.1007/s00285-020-01469-y)
Supplement: Supplementary file 1 — Supplementary material 1 (pdf 215 KB) [file 285_2020_1469_MOESM1_ESM.pdf]

SUPPLEMENTAL MATERIAL

Best Match Graphs and Reconciliation of Gene Trees with Species Trees

Manuela Geiß · Marcos E. González Laffitte · Alitzel López Sánchez · Dulce I. Valdivia · Marc Hellmuth · Maribel Hernández Rosales · Peter F. Stadler

1 Extraction of Graphs from Simulated Evolutionary Scenarios

The simulations of evolutionary scenarios (described in the main text) result in an event-labeled gene tree (T,t,σ) as well as an explicit reconciliation map $\mu : V(T) \to V(S) \cup E(S)$. From these data we have to construct the orthology graph $\Theta(T,t)$ and the RBMG $G(T,\sigma)$. This can be achieved in $O(L^2)$ time using Tarjan's off-line lowest common ancestors algorithm (Tarjan, 1979; Gabow and Tarjan, 1983) to first tabu-

Manuela Geiß
Bioinformatics Group, Department of Computer Science; and Interdisciplinary Center of Bioinformatics, University of Leipzig, Härtelstraße 16-18, D-04107 Leipzig, Germany
E-mail: manuela@bioinf.uni-leipzig.de

Maribel Hernandez-Rosales, Alitzel López
CONACYT-Instituto de Matemáticas, UNAM Juriquilla, Blvd. Juriquilla 3001, 76230 Juriquilla, Querétaro, QRO, México
E-mail: maribel@im.unam.mx, lopez.alitzel@gmail.com

Dulce I. Valdivia
Universidad Autónoma de Aguascalientes, Centro de Ciencias Básicas, Av. Universidad 940, 20131 Aguascalientes, AGS, México; Instituto de Matemáticas, UNAM Juriquilla, Blvd. Juriquilla 3001, 76230 Juriquilla, Querétaro, QRO, México.
E-mail: dulce.i.valdivia@gmail.com

Marc Hellmuth
Institute of Mathematics and Computer Science, University of Greifswald, Walther-Rathenau-Straße 47, D-17487 Greifswald, Germany; Center for Bioinformatics, Saarland University, Building E 2.1, P.O. Box 151150, D-66041 Saarbrücken, Germany
E-mail: mhellmuth@mailbox.org

Peter F. Stadler
Bioinformatics Group, Department of Computer Science; Interdisciplinary Center of Bioinformatics; German Centre for Integrative Biodiversity Research (iDiv) Halle-Jena-Leipzig; Competence Center for Scalable Data Services and Solutions; and Leipzig Research Center for Civilization Diseases, Leipzig University, Härtelstraße 16-18, D-04107 Leipzig; and Max-Planck-Institute for Mathematics in the Sciences, Inselstraße 22, D-04103 Leipzig; and Inst. f. Theoretical Chemistry, University of Vienna, Währingerstraße 17, A-1090 Wien, Austria; and Facultad de Ciencias, Universidad National de Colombia, Sede Bogotá, Colombia; Santa Fe Institute, 1399 Hyde Park Rd., Santa Fe, NM 87501, USA
E-mail: studla@bioinf.uni-leipzig.de

late $\mathrm{lca}_T(x,y)$ for all $x,y \in L$ in quadratic total time or with the help of additional data structures that then allow to answer least common ancestor queries in constant time (Harel and Tarjan, 1984; Schieber and Vishkin, 1988). As show below, it is also possible to avoid computation of the lca() function altogether.

1.1 Orthology Graphs

The orthology relation $\Theta(T,t)$ is easily constructed from the event-labeled gene tree (T,t) by a simple recursive construction. For each $v \in \widetilde{T}$ we define a graph $\Theta(v)$ recursively: if v is a leaf, then $\Theta(v)$ is the K_1 with vertex set $\{v\}$ whenever v is an extant gene and $\Theta(v) = \emptyset$, the empty graph, if v is a loss event. For inner vertices we set

$$\Theta(v) = \begin{cases} \bigotimes_{u\in \mathsf{child}(v)} \Theta(u) & \text{if } t(v) = \bullet \\ \bigcup_{u\in \mathsf{child}(v)} \Theta(u) & \text{otherwise} \end{cases} \qquad (1)$$

Since $H \bowtie \emptyset = H \cup \emptyset = H$, there is no contribution of the loss-leafs. Thus $\Theta(v)$ can be computed in exactly the same manner from the observable gene tree T. Hence, $\Theta(\rho_T) = \Theta(\rho_{\widetilde{T}}) =: \Theta$ is the orthology graph of the scenario. Note that the planted root 0_T does not appear as the last common ancestor of any two leaves in $L(T)$, hence it suffices to consider the root. Although the next result is an immediate consequence of the definition of cographs and their corresponding cotrees (Corneil *et al.*, 1981):

Lemma 1 *Let (T,t,σ) be an event-labeled, leaf-labeled tree. Then $xy \in E(\Theta(v))$ if and only if $t(\mathrm{lca}_T(x,y)) = \bullet$.*

By construction, $\Theta(u)$ is an induced subgraph of $\Theta(v)$ whenever $u \preceq_T v$. It is thus sufficient to store the binary $|L| \times |L|$ adjacency matrix of Θ. Traversing T in post-order, one sets $\Theta_{xy} = 1$, i.e., $xy \in E(\Theta)$, for all xy with $x \in L(T(u_1))$ and $y \in L(T(u_2))$ where u_1 and u_2 are distinct children of v, if and only if v is a speciation vertex. Since the pair x,y is considered exactly once, namely when $v = \mathrm{lca}(x,y)$ is encountered in the traversal of T, the total effort is $O(|L|^2)$.

1.2 Best Match Graphs

In order to compute the BMG $\vec{G}(T,\sigma)$ we associate every inner vertex v with the lists $L_r(v) := \{x \in L(T(v)) | \sigma(x) = r\}$ of leaves below v with color r. We have $L_r(v) = \bigcup_{u\in\mathsf{child}(v)} L_r(u)$ for inner vertices, while leaves are initialized with $L_r(v) = \{v\}$ if $\sigma(v) = r$, and $L_r(v) = \emptyset$ if $\sigma(v) \neq r$. Again this can be achieved in not more than quadratic time. Now define $C_{\neg s}(v) := \{u \in \mathsf{child}(v) | L_s(u) = \emptyset\}$ and $C_s(v) := \{u \in \mathsf{child}(v) | L_s(u) \neq \emptyset\}$. Best matches can be retrieved directly from these auxiliary sets:

Lemma 2 *Let u_1 and u_2 be two distinct children of some inner vertex v of the leaf-colored tree (T,σ) and let $x \in L(T(u_1))$ with $\sigma(x) = r$ and $y \in L(T(u_2))$ with $\sigma(y) = s \neq r$. Then (x,y) is a best match in (T,σ) if and only if*

$$u_1 \in C_r(v) \cap C_{\neg s}(v) \quad \text{and} \quad u_2 \in C_s(v).$$

Proof If $L_s(u_1) = \emptyset$, then there is no best match of color s for x in $L(T(u_1))$, i.e., any best match $\sigma(y') = s$ satisfies $v \preceq \text{lca}(x,y')$. From $\text{lca}(x,y) = v$ we see that (x,y) is indeed a best match. On the other hand, if $L_s(u_1) \neq \emptyset$, then there is a leaf $y' \in L_s(u_1)$ with $\text{lca}(x,y') \preceq u_1 \prec v = \text{lca}(x,y)$, and thus y is not a best match for x. □

Algorithm 1 Construction of $\vec{G}(T,\sigma)$

Require: leaf-colored tree (T,σ)
  for all leaves v of T, colors r **do**
    $L(T(v)) = \{v\}$
    if $\sigma(v) = r$ **then**
      $\ell_{vr} = 1$
    else
      $\ell_{vr} = 0$
  for all inner vertices v of T in postorder **do**
    for all $u_1, u_2 \in \mathsf{child}(v)$, $u_1 \neq u_2$ **do**
      for all $x \in L(T(u_1))$ and $y \in L(T(u_2))$ **do**
        $(x,y) \in \vec{G}(T,\sigma)$ if $\ell_{u_1\sigma(y)} = 0$
    $L(T(v)) = \bigcup_{u\in\mathsf{child}(v)} L(T(u))$
    for all $u \in \mathsf{child}(v)$, colors $r \in S$ **do**
      $\ell_{vr} = 1$ if $\ell_{ur} = 1$

This observation yields the very simple way to construct $\vec{G}(T,\sigma)$. Algorithm 1 iterates over all pairs of vertices $x, y \in L$ such that each pair is visited exactly once by considering for every interior vertex v exactly the pairs that are members of two distinct subtrees rooted at children u_1 and u_2 of v. Since $y \in L_{\sigma(y)}(u_2)$ and $x \in L_{\sigma(x)}(u_1)$ is guaranteed by construction, (x,y) is a best match if and only if $L_{\sigma(y)}(u_1) = \emptyset$ by Lemma 2. Using the precomputed binary variable ℓ_{vr} with value 1 if $L_r(v) \neq \emptyset$ and $\ell_{vr} = 0$ otherwise, this can be done in constant time $O(|L|)$. By traversing T in postorder, finally, we can compute the lists of leaves $L(v)$ on the fly. Since no subtree is revisited, there is no need to retain the $L(T(u))$ for the children, i.e., for each vertex v, the lists of its children can simply be concatenated. Similarly, the variables ℓ_{vr} can be obtained while traversing T using the fact that $\ell_{vr} = 1$ if and only if $\ell_{ur} = 1$ for at least one of its children. Hence, Algorithm 1 runs in $O(|L|^2)$ time with $O(|L|\,|S|)$ memory using a single postorder traversal of T.

The RBMG $G(T,\sigma)$ is now easily obtained from the BMG $\vec{G}(T,\sigma)$ by extracting its symmetric part. Clearly the effort for this step is also bounded by $O(|L|^2)$.

1.3 Good Quartets

We have seen in Section 6 that at least some false positive edges are identified by good quartets. A convenient way of listing all good quartets Q in $(\vec{G},\sigma)$ makes use of the *degree sequence* of $\vec{G}$, that is, the list $\alpha = ((\alpha_x^+, \alpha_x^-)|x \in V(\vec{G}))$ of pairs (α_x^+, α_x^-) where α_x^+ is the out-degree and α_x^- is the in-degree of the vertex $x \in V(\vec{G})$ and the list is ordered in positive lexicographical order. One easily checks that a good quartet contains neither a *2-switch* nor an *induced 3-cycle*, hence Q is uniquely defined by

its degree sequence $((2,1),(2,3),(2,3),(2,1))$ as a consequence of (Cloteaux *et al.*, 2014, Thm. 1). Regarding the coloring, it suffices to check that the two endpoints, that is, the vertices with indegree 1, have the same color $\sigma(u) = \sigma(x)$. This already implies $\sigma(v), \sigma(w) \neq \sigma(u) = \sigma(x)$. Since there is an edge between v and w, we also have $\sigma(v) \neq \sigma(w)$, i.e., the colors are determined up to a permutation of colors. The false positive edge is the one connecting the two vertices with outdegree 3.

2 Additional Information on Simulated Scenarios

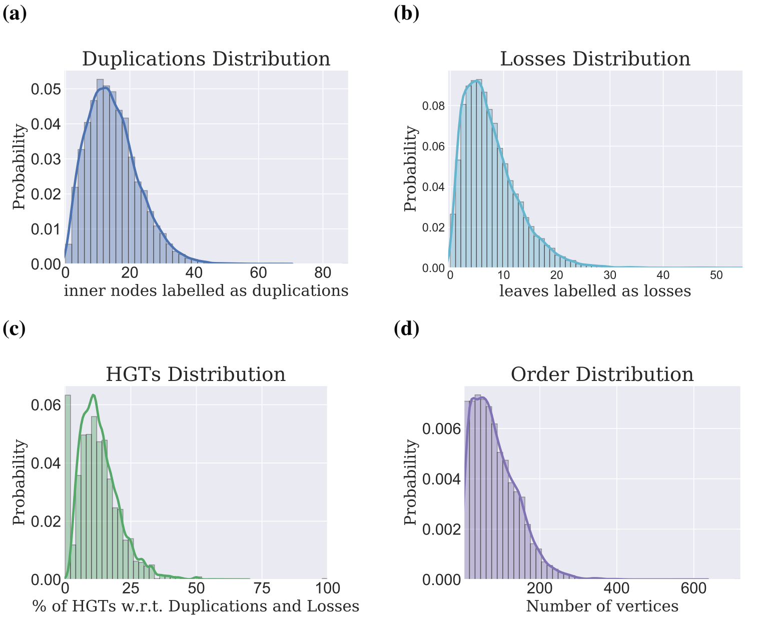

	Max.	Min.	Average
**Species**	100	3	44.5
**Extant genes**	722	3	84.62
**Speciations** (S_n)	667	2	73.46
**Duplications** (D_n)	88	0	14.66
**Losses** (L_n)	55	0	7.41
**HGT** ($H_n\%$)	100%	0%	11.76%

Fig. 1 Summary statistics of the 14,000 simulated scenarios. (a)–(c) Distributions of fraction of duplications, losses, and HGTs, respectively in the true gene trees $\widetilde{T}$. (d) Distribution of the number of extant genes in the observable gene tree T and thus the number of vertices (order) of the best match graph $G(T,\sigma)$. The spline in each panel is a kernel density estimate.

Table 1 We simulated 11 batches with different ranges for the rates of duplications, losses, and HGT (columns 3 to 5), where the rates have been varied in steps of 0.01. In each batch, we simulated for each combination of rates exactly one scenario. The second column shows the total number of scenarios for each batch.

Batch	# Scenarios	Duplication rates	Loss rates	HGT rates	# Species
1	1000	0.75 - 0.84	0.7 - 0.79	0.1 - 0.19	3-100
2	1000	0.85 - 0.94	0.85 - 0.94	0.1 - 0.19	3-100
3	1000	0.80 - 0.89	0.80 - 0.89	0.1 - 0.19	3-100
4	1000	0.70 - 0.79	0.70 - 0.79	0.1 - 0.19	3-100
5	1000	0.90 - 0.99	0.90 - 0.99	0.1 - 0.19	3-100
6	1000	0.85 - 0.94	0.75 - 0.84	0.1 - 0.19	3-100
7	1000	0.90 - 0.99	0.90 - 0.99	0.15 - 0.24	3-100
8	1000	0.90 - 0.99	0.90 - 0.99	0.15 - 0.24	3-100
9	1000	0.65 - 0.74	0.65 - 0.74	0.10 - 0.19	3-100
10	1000	0.85 - 0.94	0.75 - 0.84	0.15 -0.24	3-100
11	4000	0.75 - 0.94	0.75 - 0.94	0.15 -0.24	3-50

3 False Positive Edges in Non-Cograph 3-RBMGs

In the following, we identify further false positive orthology assignments in the RBMG based on results that we recently derived in Geiß *et al.* (2019). We start by defining a color-preserving thinness relation that has been introduced in Geiß *et al.* (2019):

Definition 1 For an undirected colored graph (G,σ) two vertices a and b are in relation S, in symbols $a\mathsf{S}b$, if $N(a) = N(b)$ and $\sigma(a) = \sigma(b)$. The equivalence class of a is denoted by $[a]$. (G,σ) is called S-thin if no two distinct vertices are in relation S.

3.1 Type (B) 3-RBMGs

Let (G,σ) be a connected S-thin 3-RBMG of Type (B). Lemma 25 of (Geiß *et al.*, 2019) then implies that (G,σ) contains an induced path $P := \langle \hat{x}_1\hat{y}\hat{z}\hat{x}_2\rangle$ with three distinct colors $\sigma(\hat{x}_1) = \sigma(\hat{x}_2) =: r$, $\sigma(\hat{y}) =: s$, and $\sigma(\hat{z}) =: t$, and $N_r(\hat{y}) \cap N_r(\hat{z}) = \emptyset$ such that the vertex sets

$$
\begin{aligned}
&L^P_{t,s} := \{y \mid \langle xy\hat{z}\rangle \in \mathcal{P}_3 \text{ for any } x \in N_r(y)\},\\
&L^P_{t,r} := \{x \mid N_r(y) = \{x\} \text{ and } \langle xy\hat{z}\rangle \in \mathcal{P}_3\} \cup \{x \mid x \in L[r], N_s(x) = \emptyset, L[s] \setminus L^P_{t,s} \neq \emptyset\},\\
&L^P_{s,t} := \{z \mid \langle xz\hat{y}\rangle \in \mathcal{P}_3 \text{ for any } x \in N_r(z)\},\\
&L^P_{s,r} := \{x \mid N_r(z) = \{x\} \text{ and } xz\hat{y} \in \mathcal{P}_3\} \cup \{x \mid x \in L[r], N_t(x) = \emptyset, L[t] \setminus L^P_{s,t} \neq \emptyset\},\\
&L^P_t := L^P_{t,s} \cup L^P_{t,r},\\
&L^P_s := L^P_{s,t} \cup L^P_{s,r}, \text{ and}\\
&L^P_* := L \setminus (L^P_t \cup L^P_s)
\end{aligned}
$$

satisfy the following conditions:

(B2.a) If $x \in L^P_*[r]$, then $N(x) = L^P_* \setminus \{x\}$,
(B2.b) If $x \in L^P_t[r]$, then $N_s(x) \subset L^P_t$ and $|N_s(x)| \leq 1$, and $N_t(x) = L^P_*[t]$,
(B2.c) If $x \in L^P_s[r]$, then $N_t(x) \subset L^P_s$ and $|N_t(x)| \leq 1$, and $N_s(x) = L^P_*[s]$
(B3.a) If $y \in L^P_*[s]$, then $N(y) = L^P_s \cup (L^P_* \setminus \{y\})$,
(B3.b) If $y \in L^P_t[s]$, then $N_r(y) \subset L^P_t$ and $|N_r(y)| \leq 1$, and $N_t(y) = L[t]$,
(B4.a) If $z \in L^P_*[t]$, then $N(z) = L^P_t \cup (L^P_* \setminus \{z\})$,
(B4.b) If $z \in L^P_s[t]$, then $N_r(z) \subset L^P_s$ and $|N_r(z)| \leq 1$, and $N_s(z) = L[s]$.

By construction, $\sigma(L^P_t) = \{r,s\}$ and $\sigma(L^P_s) = \{r,t\}$ and, as a consequence of Lemma 25 of Geiß *et al.* (2019), the sets L^P_s, L^P_t, and L^P_* form a partition of $V(G)$. Furthermore, Lemma 33 of Geiß *et al.* (2019) implies that any 3-colored induced path P of the form (r,s,t,r) that satisfies (B2.a) to (B4.b) is a good quartet w.r.t. some (T,σ) explaining a BMG $(\vec{G},\sigma)$ that contains (G,σ) as its symmetric part.

Our goal is to identify edges in (G,σ) that can cannot be present in the orthology graph Θ. To this end we extend the leaf sets L^P_*, L^P_s, L^P_t that have been introduced in Geiß *et al.* (2019) for S-thin 3-RBMGs, to general 3-RBMGs:

Definition 2 Let (G,σ) be a 3-RBMG of Type (B) with vertex set L and colors $S = \{r,s,t\}$, and $(G/\mathsf{S},\sigma_{/\mathsf{S}})$ with vertex set $\overline{L}$ be its S-thin version. We set

$$L_s^P := \{x \mid x \in L, [x] \in \overline{L}_s^P\}$$
$$L_t^P := \{x \mid x \in L, [x] \in \overline{L}_t^P\}$$
$$L_*^P := \{x \mid x \in L, [x] \in \overline{L}_*^P\}$$

if (G,σ) is of Type (B) and $(G/\mathsf{S},\sigma_{/\mathsf{S}})$ B-like w.r.t. to the induced path P.

The cases of Type (B) and (C) 3-RBMGs will be treated separately, starting with Type (B). We first need a technical result:

Lemma 3 *Let (G,σ) be a connected 3-RBMG of Type* (B) *with vertex set L, $(G/\mathsf{S},\sigma_{/\mathsf{S}})$ its* S*-thin version with vertex set $\overline{L}$, and (T,σ) a leaf-labeled tree that explains (G,σ). Moreover, let $P := \langle[\widetilde{x}_1][\widetilde{y}][\widetilde{z}][\widetilde{x}_2]\rangle$ for some good quartet $\langle\widetilde{x}_1\widetilde{y}\widetilde{z}\widetilde{x}_2\rangle$ in $\vec{G}(T,\sigma)$, and set $v := \mathrm{lca}_T(\widetilde{x}_1,\widetilde{x}_2,\widetilde{y},\widetilde{z})$. Then the leaf sets L_s^P, L_t^P, and L_*^P, where $\sigma(\widetilde{x}_1) = \sigma(\widetilde{x}_2) = r$, $\sigma(\widetilde{y}) = s$, and $\sigma(\widetilde{z}) = t$, satisfy:*

(i) $L_t^P, L_s^P \subseteq L(T(v))$,
(ii) If $L_c^P \cap L(T(v')) \neq \emptyset$ *for some* $v' \in \mathsf{child}(v)$ *and* $c \in \{s,t\}$, *then*
- *(a)* $L_{\overline{c}}^P \cap L(T(v')) = \emptyset$, *where* $\overline{c} \in \{s,t\}, \overline{c} \neq c$,
- *(b)* $\sigma(L(T(v'))) \subseteq \sigma(L_c^P)$,

(iii) $\mathrm{lca}_T(a,b) = v$ *for any* $a \in L_*^P$, $b \notin L_*^P$ *with* $ab \in E(G)$.

Proof Throughout this proof we will often use the fact that $xy \in E(G)$ if and only if $[x][y] \in E(G/\mathsf{S})$ for any $x,y \in L$ (cf. Lemma 5 of Geiß *et al.* (2019)).

Lemma 25 of Geiß *et al.* (2019) implies $[\widetilde{x}_1],[\widetilde{y}] \in \overline{L}_t^P$ and $[\widetilde{x}_2],[\widetilde{z}] \in \overline{L}_s^P$, thus, by definition, we have $\widetilde{x}_1,\widetilde{y} \in L_t^P$ and $\widetilde{x}_2,\widetilde{z} \in L_s^P$. Moreover, by Lemma 36 of Geiß *et al.* (2019), there exist distinct children $v_1,v_2 \in \mathsf{child}(v)$ such that $\widetilde{x}_1,\widetilde{y} \preceq_T v_1$ and $\widetilde{x}_2,\widetilde{z} \preceq_T v_2$. Therefore $\widetilde{y}\widetilde{z} \in E(G)$ implies $\sigma(L(T(v_1))) = \{r,s\}$; otherwise there exists a leaf $z' \in L(T(v_1)) \cap L[t]$ which implies $\mathrm{lca}_T(\widetilde{y},z') \prec_T v = \mathrm{lca}_T(\widetilde{y},\widetilde{z})$; a contradiction to $\widetilde{y}\widetilde{z} \in E(G)$. Analogously we obtain $\sigma(L(T(v_2))) = \{r,t\}$.

(i) By symmetry, it suffices to consider L_t^P in more detail, analogous arguments can then be applied to L_s^P. Let $a \in L_t^P$, or equivalently $[a] \in \overline{L}_t^P$ by definition, and suppose first $\sigma(a) = s$. Then Property (B3.b) implies $[a][\widetilde{z}] \in E(G/\mathsf{S})$. As a consequence of Lemma 5 of Geiß *et al.* (2019) we thus have $a\widetilde{z} \in E(G)$. Hence, $\widetilde{y}\widetilde{z} \in E(G)$ implies $\mathrm{lca}_T(a,\widetilde{z}) = \mathrm{lca}_T(\widetilde{y},\widetilde{z}) = v$ and thus, $a \preceq_T v$. We therefore conclude $L_t^P \cap L[s] \subseteq L(T(v))$. Now assume $\sigma(a) = r$. By Property (B2.b), we either have $N_s([a]) = \emptyset$ or there exists a vertex $y \in L[s]$ such that $[y] \in \overline{L}_t^P$ and $N_s([a]) = \{[y]\}$. In the latter case, since $[y] \in \overline{L}_t^P$ implies $y \in L_t^P$ and, in addition, it holds $L_t^P \cap L[s] \subseteq L(T(v))$, we have $y \preceq_T v$. Moreover, by (B3.b), it holds $[\widetilde{x}_2][y] \notin E(G/\mathsf{S})$, hence $\widetilde{x}_2 y \notin E(G)$. As a consequence of the latter and the fact that $[a][y] \in E(G/\mathsf{S})$ implies $ay \in E(G)$, it must hold $\mathrm{lca}_T(a,y) \prec_T \mathrm{lca}_T(\widetilde{x}_2,y) \preceq_T v$ and thus, $a \preceq_T v$. Otherwise, if $N_s([a]) = \emptyset$, then there must exist a leaf $z \in L[t]$ such that $[z] \in N_t([a])$ due to the connectedness of G/S, which is implied by the connectedness of G (cf. Lemma 5 of Geiß *et al.* (2019)). Since $[a] \in \overline{L}_t^P$, Properties (B4.a) and (B4.b) immediately imply $[z] \in \overline{L}_*^P$. Then, by

(B4.a), the edge $[\widetilde{x}_1][z]$ must be contained in G/S, thus $\widetilde{x}_1 z \in E(G)$. Since $\widetilde{x}_1, \widetilde{z} \preceq_T v$ by Lemma 36 of Geiß *et al.* (2019), it must thus hold $\mathrm{lca}_T(\widetilde{x}_1, z) \preceq_T \mathrm{lca}_T(\widetilde{x}_1, \widetilde{z}) \preceq_T v$. Therefore $\widetilde{x}_1 z, az \in E(G)$ implies $\mathrm{lca}_T(a,z) = \mathrm{lca}_T(\widetilde{x}_1, z) \preceq_T v$ and thus, $a \preceq_T v$. Hence, $L_t^P \cap L[r] \subseteq L(T(v))$, which finally implies $L_t^P \subseteq L(T(v))$.

(ii) By symmetry, it again suffices to consider the case $c = t$. Let $a \in L_t^P \cap L(T(v'))$ for some $v' \in \mathsf{child}(v)$. Note that, by (i), such a leaf a and inner vertex v' must exist. We need to distinguish the two Cases (1) $\sigma(a) = s$ and (2) $\sigma(a) = r$.

Consider first Case (1), thus in particular $s \in \sigma(L(T(v')))$. Then, as $\sigma(L(T(v_2))) = \{r,t\}$, we have $v' \neq v_2$ and thus, $\mathrm{lca}_T(a, \widetilde{z}) = v$. Hence, as $[a][\widetilde{z}] \in E(G/\mathsf{S})$ by Property (B3.b) and therefore, $a\widetilde{z} \in E(G)$, we can conclude $t \notin \sigma(L(T(v')))$ by analogous arguments as just used for showing $\sigma(L(T(v_1))) = \{r,s\}$. This implies (ii.b). Now assume, for contradiction, that there exists a leaf $x \in L(T(v')) \cap L_s^P$. Since $t \notin \sigma(L(T(v')))$ and, by definition, $s \notin \sigma(L_s^P)$, this leaf x must be of color r. Clearly, either there exists a leaf $y \in L[s]$ such that $xy \in E(G)$ or $N_s(x) = \emptyset$. In the first case, we have $[x][y] \in E(G/\mathsf{S})$ and thus, by (B2.c), $[y] \in \overline{L}_*^P$ which implies $y \in L_*^P$. In particular, as $s \in \sigma(L(T(v')))$ and $xy \in E(G)$ implies $\mathrm{lca}_T(x,y) \preceq_T \mathrm{lca}_T(x,y')$ for any $y' \in L[s]$, we can conclude $y \preceq_T v'$. Moreover, since $[\widetilde{x}_2] \in \overline{L}_s^P$, Property (B3.a) implies $[\widetilde{x}_2][y] \in E(G/\mathsf{S})$ and thus, $\widetilde{x}_2 y \in E(G)$. However, since $v' \neq v_2$, we have $\mathrm{lca}_T(x,y) \preceq_T v' \prec_T v = \mathrm{lca}_T(\widetilde{x}_2, y)$; a contradiction to $\widetilde{x}_2 y \in E(G)$. We thus conclude $N_s(x) = \emptyset$. Hence, as G is connected, there must exist a leaf z' of color t such that $xz' \in E(G)$, which implies $[x][z'] \in E(G/\mathsf{S})$. By Property (B2.c), we have $[z'] \in \overline{L}_s^P$ and therefore, (B4.b) implies $N_r([z']) = \{[x]\}$. Since $t \notin \sigma(L(T(v')))$, there is a $v'' \in \mathsf{child}(v) \setminus \{v'\}$ such that $z' \preceq_T v'' \prec_T v$. From $xz' \in E(G)$ and $\mathrm{lca}_T(x,z') = v$, we conclude that $r \notin \sigma(L(T(v'')))$. Moreover, Lemma 10 of Geiß *et al.* (2019) implies that there exist leaves $x', y' \in L(T(v'))$ with $\sigma(x') = r$ and $\sigma(y') = s$ such that $x'y' \in E(G)$. Thus, as by assumption $N_s(x) = \emptyset$, we in particular have $[x] \neq [x']$. Since $r \notin \sigma(L(T(v'')))$ and $t \notin \sigma(L(T(v')))$, it follows $x'z' \in E(G)$ and therefore, $[x'] \in N_r([z'])$; a contradiction to $N_r([z']) = \{[x]\}$. This implies (ii.a).

Now consider Case (2), i.e., $\sigma(a) = r$. We first show that $\sigma(L(T(v'))) \subsetneq \{r,s,t\}$ holds. Assume, for contradiction, that $L(T(v'))$ contains leaves $y \in L[s]$ and $z \in L[t]$. If $v' \neq v_2$, this implies $\mathrm{lca}_T(y,z) \prec_T v = \mathrm{lca}(y, \widetilde{z})$ and thus, $y\widetilde{z} \notin E(G)$ and in particular $[y][\widetilde{z}] \notin E(G/\mathsf{S})$; a contradiction to (B4.b). One analogously obtains a contradiction for the case $v' \neq v_1$; therefore $\sigma(L(T(v'))) \subsetneq \{r,s,t\}$ and we either have $\sigma(L(T(v'))) \subseteq \{r,s\}$ or $\sigma(L(T(v'))) \subseteq \{r,t\}$. If $\sigma(L(T(v'))) = \{r\}$, then it clearly holds $N(x) = N(a)$ and thus $x \in L_t^P$ for any $x \in L(T(v'))$, hence (ii.a) and (ii.b) are trivially satisfied. If $\sigma(L(T(v'))) = \{r,s\}$, then (ii.b) is trivially satisfied. Moreover, by Lemma 10 of Geiß *et al.* (2019), $L(T(v'))$ contains leaves $x' \in L[r]$ and $y' \in L[s]$ such that $x'y' \in E(G)$. Hence, we have $[x'][y'] \in E(G/\mathsf{S})$ and Property (B4.b) implies $[y'][\widetilde{z}] \in E(G/\mathsf{S})$ and thus, $y'\widetilde{z} \in E(G)$. As $\sigma(L(T(v_2))) = \{r,t\}$ and $\sigma(L(T(v'))) = \{r,s\}$, we clearly have $v' \neq v_2$ and thus, $\mathrm{lca}_T(x',y') \preceq_T v' \prec_T v = \mathrm{lca}_T(\widetilde{x}_2, y')$. Hence, $\widetilde{x}_2 y' \notin E(G)$, which implies $N([y']) \neq \overline{L}_s^P \cup (\overline{L}_*^P \setminus \{[y']\})$ since $\widetilde{x}_2 \in L_s^P$. Therefore, by Property (B3.a), we have $[y'] \notin \overline{L}_*^P$, implying $y' \notin L_*^P$. We thus conclude $y' \in L_t^P$. Hence, we can apply the argumentation of Case (1) (by substituting $a = y'$) in order to infer (ii.a).

Finally, for contradiction, assume $\sigma(L(T(v'))) = \{r,t\}$. In particular, this implies

$v_1 \neq v'$. Clearly, either there exists a leaf $y \in L[s]$ such that $ay \in E(G)$ (and thus $[a][y] \in E(G/\mathsf{S})$) or $N_s(a) = \emptyset$. In the latter case, since G is connected, there must be a leaf $z \in L[t]$ such that $az \in E(G)$ and $[a][z] \in E(G/\mathsf{S})$. In particular, as $\sigma(L(T(v'))) = \{r,t\}$, this implies $z \preceq_T v'$. By (B2.b), we have $[z] \in \overline{L}^P_*$ and thus, by (B4.a), it follows $[\widetilde{x}_1][z] \in E(G/\mathsf{S})$ implying $\widetilde{x}_1 z \in E(G)$; a contradiction since $\text{lca}_T(z,a) \preceq_T v' \prec_T v = \text{lca}_T(z,\widetilde{x}_1)$. Hence, there must exist a leaf $y \in L[s]$ such that $ay \in E(G)$. By (B2.b), we have $N_s([a]) = \{[y]\}$ and $[y] \in \overline{L}^P_t$. Then (B3.b) implies $N_r([y]) \subset \overline{L}^P_t$. It is easy to see that this implies $N_r(y) \subset L^P_t$. Since $s \notin \sigma(L(T(v')))$, there must exist a vertex $v'' \in \mathsf{child}(v) \setminus \{v'\}$ such that $y \preceq_T v'' \prec_T v = \text{lca}_T(a,y)$. One easily checks that $ay \in E(G)$ implies $r \notin \sigma(L(T(v'')))$. Together with $\sigma(L(T(v_2))) = \{r,t\}$, this implies $\text{lca}_T(\widetilde{x}_2,y) = v \preceq_T \text{lca}_T(x'',y)$ and $\text{lca}_T(\widetilde{x}_2,y) = v \preceq_T \text{lca}_T(\widetilde{x}_2,y')$ for any $x'' \in L[r]$ and $y' \in L[s]$. Thus, $\widetilde{x}_2 y \in E(G)$, which, as $\widetilde{x}_2 \in L^P_s$, contradicts $N_r(y) \subset L^P_t$. We therefore conclude that $\sigma(L(T(v'))) = \{r,t\}$ is not possible, which finally completes the proof.
(iii) Since, by definition, $V(G)$ is partitioned into L^P_s, L^P_t, and L^P_*, the leaf b must be either contained in L^P_t or L^P_s. Suppose first $b \in L^P_t$. Since $[a][b] \in E(G/\mathsf{S})$ follows from $ab \in E(G)$, Properties (B2.a), (B3.a), and (B4.a) immediately imply $\sigma(a) = t$. Moreover, by (i), there exists some $v' \in \mathsf{child}(v)$ such that $b \preceq_T v' \prec_T v$, and, by (ii.b), $\sigma(L(T(v'))) \subseteq \sigma(L^P_t) = \{r,s\}$. Hence, as $\sigma(a) = t$, we can conclude $\text{lca}_T(a,b) \succeq_T v$. Similarly, $\sigma(L(T(v'))) \subseteq \{r,s\}$ implies $\text{lca}_T(b,\widetilde{z}) = v$, thus it must hold $\text{lca}_T(a,b) \preceq_T \text{lca}_T(b,\widetilde{z}) = v$ because of $ab \in E(G)$. In summary, this implies $\text{lca}_T(a,b) = v$. Analogous arguments can be applied to the case $b \in L^P_s$.

Lemma 3 can now be used to identify a potentially very large set of edges that cannot be present in the orthology graph Θ.

Theorem 1 *Let T and S be planted trees, $\sigma : L(T) \to L(S)$ a surjective map, and μ a reconciliation map from (T,σ) to S determining an event labeling t_T on T. Moreover, let the leaf sets L^P_t, L^P_s, and L^P_* be defined w.r.t. P, which is the* S*-thin version of some good quartet of the form (r,s,t,r) in $(\vec{G},\sigma)$ with color set $S = \{r,s,t\}$. Then $t_T(\text{lca}_T(a,b)) = \square$ for any edge $ab \in E(G)$ such that $a \in L^P_\star$ and $b \notin L^P_\star$, where $\star \in \{s,t,*\}$.*

Proof Let $P = \langle [x_1][y][z][x_2] \rangle$, i.e., in particular $\sigma(x_1) = \sigma(x_2) = r$, $\sigma(y) = s$, and $\sigma(z) = t$, and let $v := \text{lca}_T(x_1,x_2,y,z)$. Then, by Lemma 36 of Geiß *et al.* (2019), there exist distinct $v_1, v_2 \in \mathsf{child}(v)$ such that $x_1, y \preceq_T v_1$ and $x_2, z \preceq_T v_2$. As $[x_1],[y] \in \overline{L}^P_t$ and $[x_2],[z] \in \overline{L}^P_s$ by Lemma 25 of Geiß *et al.* (2019) and thus, by definition, $x_1, y \in L^P_t$ and $x_2, z \in L^P_s$, Lemma 3(ii.b) in particular implies $\sigma(L(T(v_1))) = \{r,s\}$ and $\sigma(L(T(v_2))) = \{r,t\}$.
Now, if $a \in L^P_t$, $b \in L^P_s$, it follows from Lemma 3(ii.a) that $\text{lca}_T(a,b) = v$. On the other hand, if $a \in L^P_*$ and either $b \in L^P_s$ or $b \in L^P_t$, then we also have $\text{lca}_T(a,b) = v$ by Lemma 3(iii). Since $\sigma(L(T(v_1))) \cap \sigma(L(T(v_2))) = \{r\} \neq \emptyset$, we conclude from Lemma 2 that $\mu(v) \notin V^0(S)$, which implies $t_T(v) \neq \bullet$. Therefore we have $t_T(v) = \square$.

3.2 Type (C) 3-RBMGs

Let (G,σ) be a connected S-thin 3-RBMG of Type (C). Lemma 27 of (Geiß *et al.*, 2019) then implies that (G,σ) contains an induced hexagon $H := \langle \hat{x}_1 \hat{y}_1 \hat{z}_1 \hat{x}_2 \hat{y}_2 \hat{z}_2 \rangle$ with

three distinct colors $\sigma(\hat{x}_1) = \sigma(\hat{x}_2) =: r$, $\sigma(\hat{y}_1) = \sigma(\hat{y}_2) =: s$, and $\sigma(\hat{z}_1) = \sigma(\hat{z}_2) =: t$, and $|N_t(\hat{x}_1)| > 1$ such that the vertex sets

$$\begin{aligned}
L_t^H &:= \{x \mid \langle x\hat{z}_2\hat{y}_2\rangle \in \mathcal{P}_3\} \cup \{y \mid \langle y\hat{z}_1\hat{x}_2\rangle \in \mathcal{P}_3\},\\
L_s^H &:= \{x \mid \langle x\hat{y}_2\hat{z}_2\rangle \in \mathcal{P}_3\} \cup \{z \mid \langle z\hat{y}_1\hat{x}_1 \in\rangle \mathcal{P}_3\},\\
L_r^H &:= \{y \mid \langle y\hat{x}_2\hat{z}_1\rangle \in \mathcal{P}_3\} \cup \{z \mid \langle z\hat{x}_1\hat{y}_1\rangle \in \mathcal{P}_3\}, \text{ and}\\
L_*^H &:= V(G) \setminus (L_r^H \cup L_s^H \cup L_t^H)
\end{aligned}$$

satisfy the following conditions:

(C2.a) If $x \in L_*^H[r]$, then $N(x) = L_r^H \cup (L_*^H \setminus \{x\})$,
(C2.b) If $x \in L_t^H[r]$, then $N_s(x) \subset L_t^H$ and $|N_s(x)| \leq 1$, and $N_t(x) = L_*^H[t] \cup L_r^H[t]$,
(C2.c) If $x \in L_s^H[r]$, then $N_t(x) \subset L_s^H$ and $|N_t(x)| \leq 1$, and $N_s(x) = L_*^H[s] \cup L_r^H[s]$
(C3.a) If $y \in L_*^H[s]$, then $N(y) = L_s^H \cup (L_*^H \setminus \{y\})$,
(C3.b) If $y \in L_t^H[s]$, then $N_r(y) \subset L_t^H$ and $|N_r(y)| \leq 1$, and $N_t(y) = L_*^H[t] \cup L_s^H[t]$,
(C3.c) If $y \in L_r^H[s]$, then $N_t(y) \subset L_r^H$ and $|N_t(y)| \leq 1$, and $N_r(y) = L_*^H[r] \cup L_s^H[r]$,
(C4.a) If $z \in L_*^H[t]$, then $N(z) = L_t^H \cup (L_*^H \setminus \{z\})$,
(C4.b) If $z \in L_s^H[t]$, then $N_r(z) \subset L_s^H$ and $|N_r(z)| \leq 1$, and $N_s(z) = L_*^H[s] \cup L_t^H[s]$,
(C4.c) If $z \in L_r^H[t]$, then $N_s(z) \subset L_r^H$ and $|N_s(z)| \leq 1$, and $N_r(z) = L_*^H[r] \cup L_t^H[r]$.

By construction, $\sigma(L_t^H) = \{r,s\}$, $\sigma(L_s^H) = \{r,t\}$, and $\sigma(L_r^H) = \{s,t\}$ and, as a consequence of Lemma 27 of Geiß *et al.* (2019), the sets L_r^H, L_s^H, L_t^H, and L_*^H form a partition of $V(G)$. Similarly to the Type (B) case, we extend the leaf sets $L_*^H, L_r^H, L_s^H, L_t^H$ that have been introduced in Geiß *et al.* (2019) for S-thin 3-RBMGs of Type (C), to general Type (C) 3-RBMGs:

Definition 3 Let (G,σ) be a 3-RBMG of Type (C) with vertex set L and colors $S = \{r,s,t\}$, and $(G/\mathsf{S}, \sigma_{/\mathsf{S}})$ with vertex set $\overline{L}$ be its S-thin version. We set

$$\begin{aligned}
L_r^H &:= \{x \mid x \in L, [x] \in \overline{L}_r^H\}\\
L_s^H &:= \{x \mid x \in L, [x] \in \overline{L}_s^H\}\\
L_t^H &:= \{x \mid x \in L, [x] \in \overline{L}_t^H\}\\
L_*^H &:= \{x \mid x \in L, [x] \in \overline{L}_*^H\}
\end{aligned}$$

if (G,σ) is of Type (C) and $(G/\mathsf{S}, \sigma_{/\mathsf{S}})$ C-like w.r.t. to the hexagon H.

Again, we can identify edges in (G,σ) that are necessarily are false positives in the orthology graph Θ. A similar procedure as in the Type (B) case will be applied to Type (C) 3-RBMGs, again starting with an analogous technical result:

Lemma 4 *Let* (G,σ) *be a connected 3-RBMG of Type* (C) *with vertex set L,* $(G/\mathsf{S}, \sigma_{/\mathsf{S}})$ *its* S*-thin version with vertex set* $\overline{L}$*, and* (T,σ) *a leaf-labeled tree that explains* (G,σ)*. Moreover, let* $H := \langle[\widetilde{x}_1][\widetilde{y}_1][\widetilde{z}_1][\widetilde{x}_2][\widetilde{y}_2][\widetilde{z}_2]\rangle$ *for some induced hexagon* $\langle\widetilde{x}_1\widetilde{y}_1\widetilde{z}_1\widetilde{x}_2\widetilde{y}_2\widetilde{z}_2\rangle$ *in* $\vec{G}(T,\sigma)$ *with* $|N_t([\widetilde{x}_1])| > 1$ *and* $\sigma(\widetilde{x}_1) = \sigma(\widetilde{x}_2) = r$*,* $\sigma(\widetilde{y}_1) = \sigma(\widetilde{y}_2) = s$*, and* $\sigma(\widetilde{z}_1) = \sigma(\widetilde{z}_2) = t$*, and set* $v := \operatorname{lca}_T(\widetilde{x}_1,\widetilde{x}_2,\widetilde{y}_1,\widetilde{y}_2,\widetilde{z}_1,\widetilde{z}_2)$*. Then the leaf sets* L_r^H*,* L_s^H*,* L_t^H*, and* L_*^H *satisfy:*

(i) $L_r^H, L_s^H, L_t^H \subseteq L(T(v))$,

(ii) *If* $L^H_c \cap L(T(v')) \neq \emptyset$ *for some* $v' \in \mathsf{child}(v)$ *and* $c \in \{r,s,t\}$*, then*
(a) $L^H_{\overline{c}} \cap L(T(v')) = \emptyset$*, where* $\overline{c} \in \{r,s,t\}, \overline{c} \neq c$*,*
(b) $\sigma(L(T(v'))) \subseteq \sigma(L^H_c)$*,*
(iii) $\mathrm{lca}_T(a,b) = v$ *for any* $a \in L^H_*$*,* $b \notin L^H_*$ *with* $ab \in E(G)$*.*

Proof The proof of Lemma 4 closely follows the arguments leading to Lemma 3. In particular, we again use the fact that $xy \in E(G)$ if and only if $[x][y] \in E(G/\mathsf{S})$ for any $x,y \in L$ (cf. Lemma 5 of Geiß *et al.* (2019)).

By Lemma 27 of Geiß *et al.* (2019), we have $[\widetilde{x}_1],[\widetilde{y}_1] \in \overline{L}^H_t$, $[\widetilde{x}_2],[\widetilde{z}_1] \in \overline{L}^H_s$, and $[\widetilde{y}_2],[\widetilde{z}_2] \in \overline{L}^H_r$, hence $\widetilde{x}_1,\widetilde{y}_1 \in L^H_t$, $\widetilde{x}_2,\widetilde{z}_1 \in L^H_s$, and $\widetilde{y}_2,\widetilde{z}_2 \in L^H_r$. Moreover, by Lemma 39(iii) of Geiß *et al.* (2019), there exist distinct children $v_1,v_2,v_3 \in \mathsf{child}(v)$ such that $\widetilde{x}_1,\widetilde{y}_1 \preceq_T v_1$, $\widetilde{x}_2,\widetilde{z}_2 \preceq_T v_2$, and $\widetilde{y}_2,\widetilde{z}_2 \preceq_T v_3$. In particular, since $\widetilde{y}_1\widetilde{z}_1 \in E(G)$, it must hold $\sigma(L(T(v_1))) = \{r,s\}$ as otherwise there exists a leaf $z' \in L(T(v_1)) \cap L[t]$ which implies $\mathrm{lca}_T(\widetilde{y}_1,z') \prec_T v = \mathrm{lca}_T(\widetilde{y}_1,\widetilde{z}_1)$; a contradiction to $\widetilde{y}_1\widetilde{z}_1 \in E(G)$. One analogously checks $\sigma(L(T(v_2))) = \{r,t\}$ and $\sigma(L(T(v_3))) = \{s,t\}$.

(i) By symmetry, it suffices to consider L^H_t in more detail, analogous arguments can then be applied to L^H_s and L^H_r. Let $a \in L^H_t$, or equivalently $[a] \in \overline{L}^H_t$, and suppose first $\sigma(a) = r$. Then Property (C2.b) implies $[a][\widetilde{z}_2] \in E(G/\mathsf{S})$ and thus, $a\widetilde{z}_2 \in E(G)$. As $\widetilde{x}_1\widetilde{z}_2 \in E(G)$, we thus have $\mathrm{lca}_T(a,\widetilde{z}_2) = \mathrm{lca}_T(\widetilde{x}_1,\widetilde{z}_2) = v$, hence $a \preceq_T v$. We therefore conclude $L^H_t \cap L[r] \subseteq L(T(v))$. Analogously, we obtain $a \preceq_T v$ for $\sigma(a) = s$ as a consequence of Property (C3.b). In summary, we obtain $L^H_t \subseteq L(T(v))$.

(ii) Again invoking symmetry, it suffices to consider the case $c = t$. Let $a \in L^H_t \cap L(T(v'))$ for some $v' \in \mathsf{child}(v)$. First, let $\sigma(a) = r$. Then, as $r \notin \sigma(L(T(v_3)))$, we have $v' \neq v_3$ and thus, $\mathrm{lca}_T(a,\widetilde{z}_2) = v$. Hence, as $[a][\widetilde{z}_2] \in E(G/\mathsf{S})$ by Property (C2.b) and thus $a\widetilde{z}_2 \in E(G)$, we can conclude $t \notin \sigma(L(T(v')))$ using the same line of reasoning used above for showing $\sigma(L(T(v_1))) = \{r,s\}$. This implies (ii.b). Now assume, for contradiction, that there exists either (1) a leaf $x \in L(T(v')) \cap L^H_s$ or (2) a leaf $y \in L(T(v')) \cap L^H_r$.
In Case (1), since $t \notin \sigma(L(T(v')))$ and, by definition, $s \notin \sigma(L^H_s)$, this leaf x must be of color r. In particular, since L^H_s and L^H_t are disjoint, we have $x \neq a$. Hence, it must hold $s \in \sigma(L(T(v')))$ as otherwise $N(x) = N(a)$; contradicting $a \in L^H_t$, $x \in L^H_s$, and $L^H_s \cap L^H_t = \emptyset$. This immediately implies $v' \neq v_2$ because $s \notin \sigma(L(T(v_2)))$. By Property (C2.c), as $[\widetilde{y}_2] \in \overline{L}^H_r[s]$, we have $[x][\widetilde{y}_2] \in E(G/\mathsf{S})$ and thus, $x\widetilde{y}_2 \in E(G)$. However, since $s \in \sigma(L(T(v')))$, there exists a leaf $y' \preceq_T v'$ with $\sigma(y') = s$, which implies $\mathrm{lca}_T(x,y') \preceq_T v' \prec_T v = \mathrm{lca}_T(x,\widetilde{y}_2)$ because of $\widetilde{y}_2 \preceq_T v_3 \neq v'$; a contradiction to $x\widetilde{y}_2 \in E(G)$.
Hence, assume Case (2), i.e., there exists $y \in L(T(v')) \cap L^H_r$. Since $t \notin \sigma(L(T(v')))$ and, by definition, $r \notin \sigma(L^H_r)$, the leaf y must be of color s, which in particular implies $v' \neq v_2$. As $t \notin \sigma(L(T(v')))$ and $s \notin \sigma(L(T(v_2)))$, one easily checks that $y\widetilde{z}_1 \in E(G)$. However, as $y \in L^H_r$ and thus $[y] \in \overline{L}^H_r$, Property (C3.c) implies $[\widetilde{z}_1] \in \overline{L}^H_r$, hence $\widetilde{z}_1 \in L^H_r$; a contradiction since $\widetilde{z}_1 \in L^H_s$.
In summary, we conclude that $L^H_{\overline{c}} \cap L(T(v')) = \emptyset$, where $\overline{c} \in \{r,s\}$, hence (ii.a) is satisfied for $c = t$. Analogous arguments can be used to demonstrate that properties (ii.a) and (ii.b) are also satisfied for $\sigma(a) = s$.

(iii) Since, by definition, $V(G)$ is partitioned into L_r^H, L_s^H, L_t^H, and L_*^H, the leaf b must be either contained in L_r^H, L_s^H, or L_t^H. Suppose first $b \in L_t^H$. Then, since $[a][b] \in E(G/\mathsf{S})$ follows from $ab \in E(G)$, Properties (C2.a), (C3.a), and (C4.a) immediately imply $\sigma(a) = t$. Moreover, by (i), there exists some $v' \in \mathsf{child}(v)$ such that $b \preceq_T v' \prec_T v$ and, by (ii.b), $\sigma(L(T(v'))) \subseteq \sigma(L_t^H) = \{r,s\}$. Hence, as $\sigma(a) = t$, we can conclude $\mathrm{lca}_T(a,b) \succeq_T v$. Similarly, $\sigma(L(T(v'))) \subseteq \{r,s\}$ implies $\mathrm{lca}_T(b,\tilde{z}_1) = v$, thus it must hold $\mathrm{lca}_T(a,b) \preceq_T \mathrm{lca}_T(b,\tilde{z}_1) = v$ because of $ab \in E(G)$. In summary, this implies $\mathrm{lca}_T(a,b) = v$. Analogous arguments can be applied to the cases $b \in L_s^H$ and $b \in L_r^H$.

Similar to Type (B) 3-RBMGs, we use Lemma 4 to finally identify false positive edges.

Theorem 2 *Let T and S be planted trees, $\sigma : L(T) \to L(S)$ a surjective map, and μ a reconciliation map from (T,σ) to S determining an event labeling t_T on T. Moreover, let the leaf sets L_r^H, L_s^H, L_t^H, and L_*^H be defined w.r.t. H, which is the* S*-thin version of some hexagon $H' = \langle x_1y_1z_1x_2y_2z_2\rangle$ of the form (r,s,t,r,s,t) and $|N_t(x_1) > 1|$ in $(\vec{G},\sigma)$ with color set $S = \{r,s,t\}$. Then $t_T(\mathrm{lca}_T(a,b)) = \square$ for any edge $ab \in E(G)$ such that $a \in L_\star^H$ and $b \notin L_\star^H$, where $\star \in \{r,s,t,*\}$.*

Proof Let $v := \mathrm{lca}_T(x_1,x_2,y_1,y_2,z_1,z_2)$. Again, we have $[x_1],[y_1] \in \overline{L}_t^H$, $[x_2],[z_1] \in \overline{L}_s^H$, and $[y_2],[z_2] \in \overline{L}_r^H$ by Lemma 27 of Geiß *et al.* (2019) and thus, $x_1,y_1 \in L_t^H$, $x_2,z_1 \in L_s^H$, $y_2,z_2 \in L_r^H$. Moreover, by Lemma 39(iii) of Geiß *et al.* (2019), there exist distinct $v_1,v_2,v_3 \in \mathsf{child}(v)$ such that $x_1,y_1 \preceq_T v_1$, $x_2,z_1 \preceq_T v_2$, and $y_2,z_2 \preceq_T v_3$. As $x_1,y_1 \in L_t^H$, $x_2,z_1 \in L_s^H$, $y_2,z_2 \in L_r^H$, Lemma 4(ii.b) in particular implies $\sigma(L(T(v_1))) = \{r,s\}$, $\sigma(L(T(v_2))) = \{r,t\}$, and $\sigma(L(T(v_3))) = \{s,t\}$.
Now, if $a \in L_c^H$, $b \in L_{\overline{c}}^H$, where $c = \{r,s,t\}$ and $\overline{c} \in \{r,s,t\}, \overline{c} \neq c$, it follows from Lemma 4(ii.a) that $\mathrm{lca}_T(a,b) = v$. On the other hand, if $a \in L_*^H$ and $b \in L_c^H$, then we also have $\mathrm{lca}_T(a,b) = v$ by Lemma 4(iii). Since $\sigma(L(T(v_i))) \cap \sigma(L(T(v_j))) \neq \emptyset$ for $1 \leq i < j \leq 3$, we conclude from Lemma 2 that $\mu(v) \notin V^0(S)$, which implies $t_T(v) \neq \bullet$. Therefore we have $t_T(v) = \square$.

References

Cloteaux B, LaMar MD, Moseman E, Shook J, 2014. Threshold digraphs. J Res Natl Inst Standards Technology 119:227–234.

Corneil DG, Lerchs H, Steward Burlingham L, 1981. Complement reducible graphs. Discr Appl Math 3:163–174.

Gabow HN, Tarjan RE, 1983. A linear-time algorithm for a special case of disjoint set union. In: Proceedings of the 15th ACM Symposium on Theory of Computing (STOC), (pp. 246–251). New York, NY: ACM.

Geiß M, Hellmuth M, Stadler PF, 2019. Reciprocal best match graphs. Journal of Mathematical Biology DOI 10.1007/s00285-019-01413-9.

Harel D, Tarjan RE, 1984. Fast algorithms for finding nearest common ancestors. SIAM J Computing 13:338–355.

Schieber B, Vishkin U, 1988. On finding lowest common ancestors: simplification and parallelization. SIAM J Computing 17:1253–1262.

Tarjan RE, 1979. Applications of path compression on balanced trees. J ACM 26:690–715.
